# Gradient light interference microscopy for 3D imaging of unlabeled specimens

**DOI:** 10.1038/s41467-017-00190-7

**Published:** 2017-08-08

**Authors:** Tan H. Nguyen, Mikhail E. Kandel, Marcello Rubessa, Matthew B. Wheeler, Gabriel Popescu

**Affiliations:** 1 Department of Electrical and Computer Engineering, University of Illinois at Urbana-Champaign, Champaign, IL 61801 USA; 2 Carl R. Woese Institute for Genomic Biology, University of Illinois at Urbana-Champaign, Champaign, IL 61801 USA

## Abstract

Multiple scattering limits the contrast in optical imaging of thick specimens. Here, we present gradient light interference microscopy (GLIM) to extract three-dimensional information from both thin and thick unlabeled specimens. GLIM exploits a special case of low-coherence interferometry to extract phase information from the specimen, which in turn can be used to measure cell mass, volume, surface area, and their evolutions in time. Because it combines multiple intensity images that correspond to controlled phase shifts between two interfering waves, gradient light interference microscopy is capable of suppressing the incoherent background due to multiple scattering. GLIM can potentially become a valuable tool for in vitro fertilization, where contrast agents and fluorophores may impact the viability of the embryo. Since GLIM is implemented as an add-on module to an existing inverted microscope, we anticipate that it will be adopted rapidly by the biological community.

## Abstract (web summary)

Challenges in biological imaging include labeling, photobleaching and phototoxicity, as well as light scattering. Here, Nguyen et al. develop a quantitative phase method that uses low-coherence interferometry for label-free 3D imaging in scattering tissue.

## Introduction

It has become increasingly clear that understanding morphogenesis and disease requires three-dimensional (3D) tissue cultures and models^1^. Effective 3D imaging techniques, capable of reporting on subcellular as well as multicellular scales, in a time-resolved manner, are crucial for achieving this goal^2^. Although the light microscope has been the main tool of investigation in biomedicine for four centuries, the current requirements for 3D imaging pose new, difficult challenges. Owing to their insignificant absorption in the visible spectrum, most living cells exhibit very low contrast under light microscopy. As a result, fluorescence microscopy has become the main tool of investigation in cell biology^3^. Due to the significant progress in designing fluorescence tags, structures in the cell can now be imaged with high specificity.

More recently, super-resolution microscopy methods based on fluorescence have opened new directions of investigation, toward nanoscale subcellular structure^4^. However, fluorescence imaging is subject to several limitations. Absorption of the excitation light may cause the fluorophore to irreversibly alter its molecular structure and stop fluorescing. This process, known as photobleaching, limits the time interval over which continuous imaging can be performed^5^. The excitation light is typically toxic to cells, a phenomenon referred to as phototoxicity^6^. Overcoming these limitations becomes extremely challenging^7^^,^^8^ when imaging thick objects over an extended period of time^9, 10^ as acquiring data over the time and the axial dimension increases exposure of the specimen to the excitation light, lowering its viability. Confocal^11^ and two-photon fluorescence microscopy^12^ have been the mainstay tools of imaging thick 3D specimens. Although these methods can provide excellent sectioning through tissue, due to the focused, short wavelength excitation, the amount of power required may be harmful. Thus, recent advances in light sheet microscopy were dedicated specifically to reducing phototoxicity and photobleaching^13–16^.

Label-free microscopy provides an alternative solution to overcoming these limitations, albeit at the expense of molecular specificity. Two classical methods are phase contrast (PC) microscopy^17^ and differential interference contrast (DIC) microscopy^18^. The contrast in these methods is generated by visualizing the modifications of the wavefront when light propagates through the sample. Unfortunately, both PC and DIC are qualitative—that is to say, they do not measure the wavefront deformation quantitatively, and the image recorded on the detector is often substantially different from the scattering potential of the object. This deformation is characterized by a spatially dependent phase shift, defined as *ϕ*(**r**) = (2*π*/*λ*_o_)*h*(**r**)Δ*n*(**r**), where *λ*_o_ is the central wavelength of the illumination, *h*(**r**) and Δ*n*(**r**) are the sample thickness and refractive index difference, both evaluated at the transverse coordinate **r**, respectively.

Quantitative phase imaging (QPI) is an approach focused precisely on quantifying this phase shift^19^. Along these lines, Cogswell et al.^20^ proposed DIC with geometrically-induced phase shifting, applied for two-dimensional (2D) imaging. DIC with two orthogonal shear directions has been used to obtain 2D quantitative phase images^21, 22^, whereas Mehta et al.^23^ reported a partially coherent model for DIC 2D imaging. Shribak et al.^24^ used liquid crystal modulators to change the polarization directions and phase shift modulation. This setup allows them to record the 2D phase-gradient information at two orthogonal directions and reconstruct the optical phase front.

QPI has recently gained significant scientific interest, especially in the biomedical field^19^, thanks to several advancements. For example, common-path interferometry replaced traditional interferometry for better stability and sensitivity^25–28^. Low temporal coherence illumination methods significantly improve image resolution when suppressing speckles^29–32^. An interesting direction of study is using QPI to extract scattering information from extremely weakly scattering objects^33^. This approach is referred to as Fourier transform light scattering (FTLS), a spatial analog to Fourier transform (infrared) spectroscopy^34^. The idea is that the knowledge of amplitude and phase of an image field allows us to numerically propagate that field to any plane, including the far field, where angular scattering measurements are typically performed. For weakly scattering objects such as live cells, it is much more signal-effective to perform the measurement at the image plane, where all scattering angles overlap at each point, rather than measuring angle-by-angle in the far field. As a result, QPI can be used to solve inverse scattering problems and extract the 3D structure of inhomogeneous objects^35^. Three-dimensional information of the specimen is accessible by measuring the phase across multiple angles of the illumination or axial specimen positions^36–39^. An equivalent approach is fixing the illumination direction while rotating the sample to obtain phase maps from different viewing angles. For example, see Merola et al.^40^ Lens-free holography method is also used to obtained tomographic information on a chip by combining with multiple illumination angles^41^.

However, imaging optically thick, multiple scattering specimens is still challenging for any optical method, including QPI. The fundamental obstacle is that multiple scattering generates an incoherent background, which ultimately degrades the image contrast. An imaging method dedicated to imaging these thick specimens must include a mechanism to subdue the multiple scattering backgrounds and exhibit strong sectioning to suppress the out-of-focus light. To overcome these challenges, here we introduce a new QPI method, referred to as gradient light interference microscopy (GLIM). GLIM combines DIC microscopy with low-coherence interferometry and holography. In GLIM, the two interfering fields are identical except for a small transverse spatial shift. This geometry ensures that the two fields suffer equal degradation due to multiple scattering. By accurately controlling the phase shift between the two waves, we acquire multiple intensity images, which have the same incoherent background, but different coherent contributions. As a result, GLIM rejects much of the multiple scattering contributions and yield high contrast of thick objects. Furthermore, the illumination condenser aperture is fully open, which lands GLIM very strong optical sectioning. GLIM can provide tomographic imaging of both thin samples, for example, single cells, and thick specimens, such as multicellular systems. Below, we present the principle of GLIM operation, validation results on test samples, and time-resolved tomography of cells in culture, as well as embryo development.

## Results

### GLIM principle

**Fig. 1:**
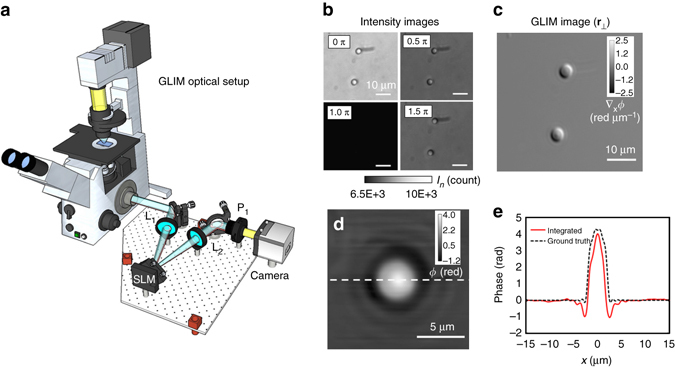
Optical setup and working principle of GLIM. **a** GLIM optical setup. The GLIM module is designed as an add-on module connected to the output port of an inverted microscope. This module shifts the phase of one polarization component using an SLM while keeping the other unmodified. Interference patterns generated by these two components are recorded and transferred to a computer for phase-gradient extraction. **b** Four frames are acquired by the GLIM module, one for each phase shift applied by the SLM. Using these images, we obtain the phase-gradient map and integrate it along the direction of the shift to recover the quantitative phase map. **c** Extracted quantitative-gradient map of two 3 µm polystyrene beads immersed in oil. **d** Integrated phase map of a 4.5 µm polystyrene microbeads at NA_con_ = 0.09. **e** Cross-sections of the reconstructed phase and the computed ground truth (*black dashed curve*) taking into account blurring due to diffraction (Eq. (3))

GLIM is an add-on module to a commercial DIC microscope as shown in Fig. 1a. Via a Wollaston prism, a typical DIC microscope generates two replicas of the image field, cross-polarized, shifted transversely by a distance smaller than the diffraction spot. We removed the analyzer that normally renders the two polarizations parallel in DIC and, instead, let the fields enter the GLIM module. These fields are spatially Fourier transformed by the lens L_1_ at its back focal plane. A spatial light modulator (SLM), placed at this plane with its active axis aligned to the polarization direction of one field, retards its phase by *ϕ*_*n*_ = *nπ*/2 with *n* = 0, 1, 2, 3, and leaves the other field unmodified. Both fields are Fourier-transformed again by lens L_2_ to generate the image at the camera plane. A linear polarizer, P_1_, is aligned at 45° with respect to both polarizations to render them parallel. The resulting field at the detector is a coherent superposition of these two fields, namely,

1 $${U_n}\left( {\bf r} \right) = U\left( {\bf r} \right) + U\left( {{\bf r} + \delta {\bf r}} \right) {{\rm e}^{i{\phi _n}}},$$

where $\delta {\bf{r}} = \delta x{{\hat {\bf x}}}$ is the spatial offset between the two fields and *U* is the image field. The intensity at each phase shift, *I*_*n*_(**r**) = |*U*_*n*_(**r**)|^2^, can be written as

2 $${I_n}\left( {\bf{r}} \right) = I\left( {\bf{r}} \right) + I\left( {{\bf{r}} + \delta {\bf{r}}} \right) + 2\left| {\gamma \left( {{\bf{r}},\delta {\bf{r}}} \right)} \right|cos\left[ {\phi \left( {{\bf{r}} + \delta {\bf{r}}} \right) - \phi \left( {\bf{r}} \right) + {\phi _n}} \right],$$

where *I*(**r**) and *ϕ*(**r**) are, respectively, the intensity and phase of the image field, and *γ* is the mutual intensity or the temporal cross-correlation function at zero-delay between these two fields, *γ*(**r**,*δ* **r**) = 〈*U* ^*^(**r**)*U*(**r** + *δ* **r**)〉_*t*_. The quantity *ϕ*_*n*_ = *nπ*/2 is the modulated phase offset between the two fields, externally controlled by the SLM. From the four intensity images, *I*_*n*_, with *n* = 0, 1, 2, 3 (Fig. 1b), we are able to solve for *I*(**r**), |*γ*(**r**,*δ* **r**)|, and Δ*ϕ*(**r**) = *ϕ*(**r** + *δ* **r**)−*ϕ*(**r**). These data render quantitatively the gradient of the phase along the direction of the shift (Fig. 1c), ∇_*x*_ *ϕ*(**r**) ≈ Δ*ϕ*(**r**)/*δx*. Details on the optical setup, procedures for extracting the phase gradient and estimating *δx* can be found in the “Methods” section. Before running the experiments, the SLM needs a one-time calibration to ensure proper phase modulation. The calibration procedure and pixel-to-pixel variation of the SLM are described in details in Supplementary Notes 1, 2, and Supplementary Fig. 1.

### QPI using GLIM

To demonstrate the capability of GLIM to extract quantitatively the phase gradient, we imaged 4.5 ± 5% µm polystyrene micro-beads (Polysciences Inc.), with a refractive index value of 1.59 at the central wavelength. The beads are immersed in immersion oil (Zeiss Inc.) with a refractive index value of 1.518 to generate a total phase shift of 3.87 radians. Figure 1c shows the measured phase gradient at NA_con_ = 0.09, where the subscript con stands for condenser. Given the phase gradient, ∇_*x*_ *ϕ*, one can integrate along the gradient direction to get phase value, *ϕ*(**r**), using

3 $$\phi \left( {x,y} \right) = \mathop {\int}\limits_0^x {\left[ {{\nabla _x}\phi \left( {x',y} \right)} \right]} \,{\rm{d}}x' + \phi \left( {0,y} \right),$$

where *ϕ*(0,*y*) is the initial value, which can be obtained with some prior knowledge of the specimen. For example, if (0,*y*) is a background location, the phase *ϕ*(0,*y*) should be set to 0 radians. Figure 1d shows the quantitative phase map, *ϕ*(**r**), and Fig. 1e displays a line profile through the center of the bead. Note that our integration result matches well with the expected ground truth, where optical diffraction is taken into account.

### GLIM imaging of cell cultures

Due to the low phototoxicity, absence of photobleaching, and easy sample preparation, transmitted light modalities appear to be ideal for studying cell growth and proliferation^42^. Yet, such assays are most frequently conducted with the aid of labels. Although specificity granted by external markers is crucial for certain applications, quantifying cell growth over longer timescales remains a challenge^43^. It has been known for some time that indicators of cell proliferation do not have equal growth^44^. More recently, new approaches have been demonstrated using vibrating hollow cantilevers to weigh cell passing through^45^. This method is limited to non-adherent cells. To expand the principle of this measurement to adherent cells, a method based on vibrating pedestals was demonstrated, at the expense of mass sensitivity^46^.

**Fig. 2:**
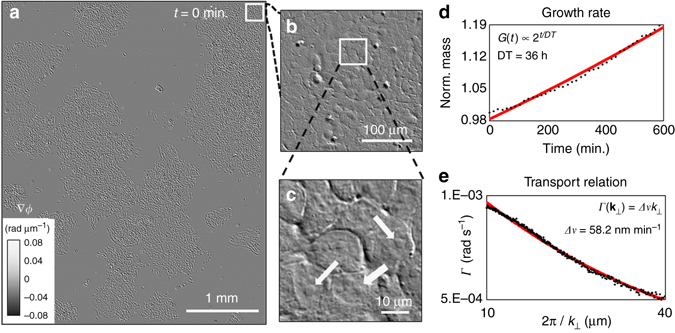
Time-lapse GLIM imaging of a HeLa cell culture. **a** The image sequence is composed of 16 × 20 mosaic tiles, each of 280 × 277 μm^2^ area with an acquisition performed every 16 min. **b** Image of each mosaic tile of a small region in **a**. **c** A zoomed in region of **b**. The resulting image is well suited to observe cellular morphology, in particular, cells nucleoli are well represented (*white arrows* in **c**). The *same colorbar* applies for all figures from **a**–**c**. From the GLIM image, it is possible to measure the mass doubling time, *DT*. **d** Measured normalized mass profile (*black dotted*) and an exponentially fitted one (*red*). **e** The slope measurement Γ(**k**_⊥_) of the temporal autocorrelation function as a function of **k**_⊥_. *Red profile*: fitting curve of Γ(**k**_⊥_) using the expression in Eq. (6). We estimated variance of the advection velocities corresponding to active transport, Δ*ν* ≈ 58.2 nm min^−1^. The contribution of diffusion motion is negligible from the fit

We show that GLIM is able to quantify the growth and proliferation of large populations of adherent cells over extended periods of time. Specifically, we can characterize the culture by extracting parameters such as single cell mass, volume, surface area, while simultaneously measuring the intracellular transport on timescales associated with the cell cycle. Supplementary Note 3 provides details on 2D image formation in GLIM. Figure 2a shows scanning GLIM data of HeLa cells in culture over a 4.48 × 5.54 mm^2^ field of view. This image is a mosaic consisting of 16 × 20 individual frames, imaged by a 40×/0.75 NA objective and a condenser aperture adjusted to NA_con_ = 0.32. Figure 2b and c shows magnified views at different scales for a region denoted by the *white box* in Fig. 2a. The acquisition took ~3 min for each of the 16 × 20 mosaics, which was assembled into a time-lapse sequence following the procedure outlined in the “Methods” section, Supplementary Note 4, and Supplementary Figs. 3 and 4. The GLIM image (Fig. 2c) provides a quantitative phase-gradient map at the spatial resolution of the objective, clearly showing fine structures such as nucleoli pointed by *white arrows*. We acquired 38 such large fields of view, over a 10 h time interval (see the Supplementary Movie 1 for a time-lapse sequence).

To measure growth rates, the phase values are obtained by integrating the phase gradient at each frame in the time-lapse sequence using Eq. (3). These phase values are used to calculate the cell dry mass, using the linear relationship between the optical path-length map of a cell and its dry mass density, *ρ*, see Barrer^47^. The *black dotted* profile in Fig. 2d shows the normalized dry mass to that at time *t* = 0. Fitting this profile to the function 2^*t*/DT^, where DT is the mass doubling time constant, we found that DT ≈ 36 h. Interestingly, this time is approximately 50% longer than the typical cell count-doubling time as cells can divide without doubling in mass^48^. The fitted profile is shown in *red* in Fig. 2d.

To investigate the physics of cellular mass transport, we use the dispersion phase spectroscopy (DPS) method^49^. This approach is powerful in extracting spatiotemporal fluctuation information from time-lapse sequence of phase maps, as it requires no manual tracing, making it well suited for fully automated applications^50^. In DPS, one computes the dispersion relation of the dynamic system, connecting the spatial and temporal frequencies. The behavior of this dispersion curve informs on the nature of the transport (such as, comparisons of diffusion vs. deterministic dominated transport) and numerical fits yield the diffusion coefficient, and the width of the velocity distribution. Let us start by describing the dry mass-density fluctuation, *ρ*(**r**_⊥_,*t*), via an advection-diffusion equation, namely,

4 $$D{\nabla ^2}\rho \left( {{{\bf{r}}_ \bot },t} \right) - {\bf{v}}.\nabla \rho \left( {{{\bf{r}}_ \bot },t} \right) - \partial \rho \left( {{{\bf{r}}_ \bot },t} \right)/\partial t = 0,$$

where **r**_⊥_ = (*x*,*y*) is the 2D coordinate vector, **v** is the advection velocity vector, and *D* is the average diffusion coefficient. Using this equation, we obtain the temporal autocorrelation function evaluated at each spatial frequency, **k**_⊥_, and temporal delay, *τ*, defined as $g\left( {{{\bf{k}}_ \bot },\tau } \right) = {\left\langle {\tilde \rho \left( {{{\bf{k}}_ \bot },t} \right){{\tilde \rho }^*}\left( {{{\bf{k}}_ \bot },t + \tau } \right)} \right\rangle _t}/{\left\langle {\tilde \rho \left( {{{\bf{k}}_ \bot },t} \right){{\tilde \rho }^*}\left( {{{\bf{k}}_ \bot },t} \right)} \right\rangle _t}$. Here, $\tilde \rho \left( {{{\bf{k}}_ \bot },t} \right) = {{\rm F}_{{{\bf{k}}_ \bot }}}\left[ {\rho \left( {{{\bf{r}}_ \bot },t} \right)} \right]$ is the 2D spatial Fourier transform of the dry mass density. We calculated *g*(**k**_⊥_,*τ*) directly from the phase gradient ∇*ϕ* instead of the integrated phase *ϕ* (Supplementary Note 5), namely

5 $$g\left( {{{\bf{k}}_ \bot },\tau } \right) = \exp \left( {i{{\bf{v}}_{\rm{o}}}.{{\bf{k}}_ \bot }\tau } \right)\exp \left[ { - \left( {\Delta v{k_ \bot } + Dk_ \bot ^2} \right)\tau } \right],$$

where **v**_o_ is the mean and Δ*v* the standard deviation of the velocity distribution. At each transverse spatial frequency, **k**_⊥_, one can fit the measurement of *g*(**k**_⊥_,*τ*) using Eq. (5) to estimate Δ*v* and the diffusion coefficient, *D*. We found that **v**_o_ is negligible for the duration of the experiment or that no dominant velocity vector, **v**_o_,was detected in our data. The decay rate of *g*(**k**_⊥_,*τ*) at each spatial mode **k**_⊥_ satisfies

6 $$\Gamma \left( {{{\bf{k}}_ \bot }} \right) = \Delta v{k_ \bot } + Dk_ \bot ^2.$$

The first term in Eq. (6) dictates the active transport, whereas the remaining term characterizes diffusion. The *black dot* profile in Fig. 2e is our measured Γ(**k**_⊥_). Fitting this profile to Eq. (6), we found that active transport dominated on cellular scales (10–50 μm), with a spread in velocities of Δ*ν* ≈ 58 nm min^−1^. Fitted profiles are shown in *red*.

### Tomography of single cells using GLIM

**Fig. 3:**
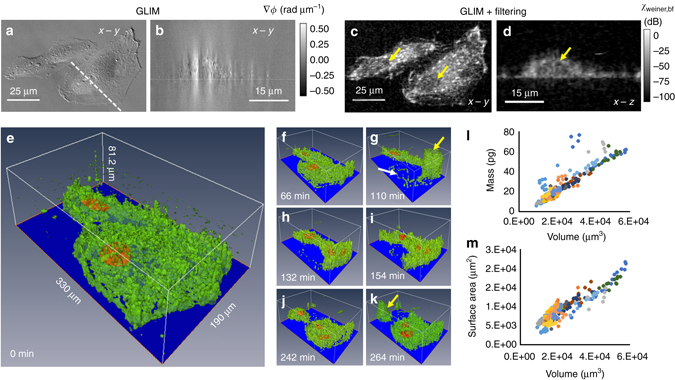
GLIM time-lapsed tomography of a HeLa cell culture. **a** *x*−*y* cross-section and **b** *x*−*z* cross-section of the GLIM phase-gradient measurement. *Dashed line* denotes the locations of the cross-section. **c** *x*−*y* cross-section and **d** *x*−*z* cross-section after spatial filtering. **e**–**k** Rendering of cell tomography results at different time, as indicated. Here, the cell nuclei are segmented and displayed in orange. The cell membranes are in *green*. **l** Mass vs. volume and **m** area vs. volume measurements extracted from the GLIM tomography data. *Data points* with the *same color* belong to the same cell but were acquired a different time points

As a result of the high numerical aperture of the illumination, GLIM has strong sectioning capabilities, which yields tomographic imaging of both thin and thick samples. We apply GLIM tomography to a 30% confluence HeLa cell culture over 7.7 h. Seven fields of view (FOVs) were imaged using a 63×/1.4 NA objective with a spatial sampling rate of 10.8 pixels µm^−1^. Each FOV was scanned every 22 min. For each time point, the sample is scanned over a total depth of 28 µm with a step size of Δ*z* = 0.07 µm. Figures 3a and b show the *x*−*y* and *x*−*z* cross-sections of the GLIM measurement, namely the quantitative phase gradient ∇*ϕ*. To remove the background due to weak sectioning at small scattering angles, we perform a spatial high-pass filtering operation, as described in the “Methods” section. Figures 3c and d show the corresponding *x*−*y* and *x*−*z* cross-sections of the filtering, with the *yellow arrows* pointing to the locations of the nucleus. Clearly, the *x*−*z* cross-section of the tomograms shows significant improvements in depth sectioning. Compared to the phase-gradient image, ∇_*x*_ *ϕ*, this cross-section has no diffraction streaks or shadow artifacts, while preserving clear cell boundaries. Figures 3e–k show the GLIM tomograms obtained via filtering, at seven different time points. The cell nuclei were segmented and shown in *orange*, whereas the cell membranes displayed in *green* using isosurface rendering. The rendered images clearly show how the 3D shape of the cell changes over time. It can be further seen that during mitosis (the 110 and 264 min. frames), the cells assumed a spherical shape (pointed by *yellow arrows* in Fig. 3g). Also, at the 110-min point, while forming a mitotic sphere, the cells appear to leave behind biomass (*white arrow* in Fig. 3g) that is adherent to the substrate, consistent with previous observations^51^. Rendered images for the whole time series can be found in Supplementary Movie 2.

Thanks to the depth sectioning of GLIM, we can automatically segment different cells in the volume of interest (Supplementary Note 6; Supplementary Fig. 5) to compute several parameters of each cell and study their temporal evolution. An example of GLIM tomograms over a full cell cycle can be found in Supplementary Fig. 6. Figure 3l shows the dry mass (Supplementary Note 7; Supplementary Fig. 7) vs. volume for several different cells during a 21-h window. Each point in these plots corresponds to one cell at one time point. These results (also those from Supplementary Note 8) show that, for the most part, the points align along a straight line we found that the points deviating from this line correspond to cells going through mitosis. Meanwhile, the surface area vs. volume relation shown in Fig. 3m is essentially linear with slightly different slopes for different cells over the whole cell cycle.

### GLIM investigation of embryo viability

The Centers for Disease Control and Prevention (CDC) report from 2014 shows that 208,768 Assisted Reproduction Technology (ART) cycles were performed with 57,332 live births^52^. As the numbers indicate, the percentage of live births from these procedures is still rather low. One reason is the lack of objective and accurate evaluation of embryo quality and viability before transfer. Morphological assessment is currently the main method used to determine embryo viability during in vitro fertilization (IVF) cycles. However, studies have shown that the predictive power of the typical day 2 and 3 assessment of morphological parameters has remained low^53–55^. Various noninvasive analytical tools have recently been used for noninvasive prediction of embryonic potential^56–60^. One such tool has been the development of quantitative techniques for the non-invasive assessment of embryo metabolism, and its value as a predictor of embryo viability is the subject of ongoing investigations^61^. But currently, visual observation remains the most used and reliable method. With the improvement of microscopy, it is possible to follow embryo development in real time, and it has been established that morphokinetic parameters can be used to select embryos for higher viability^62^. One of the most important microscopy techniques is transmission electronic microscopy (TEM), which is considered by many the main tool for intracellular evaluation. The main problem with using TEM for embryo evaluation is that the sample preparation kills the embryo^63^. Therefore, although this type of microscopy can be considered an important tool for research, it has little value for routine IVF procedures. Other techniques used to evaluate the embryo quality include confocal microscopy^64^ and two-photon imaging^65^. Using these fluorescence methods, the sample must be tagged to be evaluated, which can be detrimental to embryo survival.

Due to its sectioning capabilities, GLIM can be used to perform tomography on optically thick specimens such as embryos. For this demonstration, we used bovine embryos, prepared as described in the Supplementary Methods. In a single experiment, we imaged 60 bovine embryos, starting at 12 h after fertilization, sampling every 30 min, over a 7-day period, using a 40×/0.75 NA objective. The embryo thicknesses are within 250–300 µm. Supplementary Movies 3 and 4 illustrate the high contrast that GLIM yields even in these challenging, multiple scattering samples. For example, the lipid droplets, prominent in bovine embryos can be clearly identified. Their contrast switches from dark to bright as they pass through the focus. Our results show that the embryo internal dynamics changes completely when the embryo dies. Specifically, the internal mass transport halts almost entirely, which suggest either a large increase in viscosity of the material or that the dynamic transport is mostly due to molecular motors, which stop in dead cells. GLIM can be a valuable tool for IVF because it provides an intrinsic marker to predict viability in advance. Toward this goal, we developed a dynamic index marker (DIM), based on the GLIM data. This metric is computed from the phase difference Δ*ϕ*(**r**_⊥_,*t*) and the mutual intensity *γ*(**r**_⊥_, *δ*r, *t*) at each time point *t*. To measure morphological changes, we compute the time derivative of *γ*, that is *γ*′_*t*_(**r**_⊥_) = d*γ*(**r**_⊥_, *δ*r, *t*)/d*t*. On the basis of *γ*′_*t*_, we calculate the spatial cumulative distribution function (CDF) of the time derivative images ${F_t}\left( x \right) = P\left\{ {{{\left| {{{\gamma '}_t}\left( {{{\bf{r}}_ \bot }} \right)} \right|}_{{r_ \bot } \in {\rm{FOV}}}} < \,x} \right\},$ which is a probability that the amplitude of the time derivative is less than a value *x*. The cut-off difference (distance in *x*) at 10 % and 90 % of the CDF for each time point *t* is defined as ${D_t} = \arg {\min _{{x_1}}}\left[ {F\left( {{x_1}} \right)  >0.9} \right] - \arg {\min _{{x_2}}}\left[ {F\left( {{x_2}} \right)  >0.1} \right].$ Finally, we define the DIM as the relative ratio between *D*_*t*_ and its maximum over the imaging duration, max_*t*_ (D_*t*_), namely

7 $${\rm{DIM}}\left( t \right) = \left[ {{D_t}/{{\max }_{{t_1}}}\,\left( {{D_{{t_1}}}} \right)} \right].$$

**Fig. 4:**
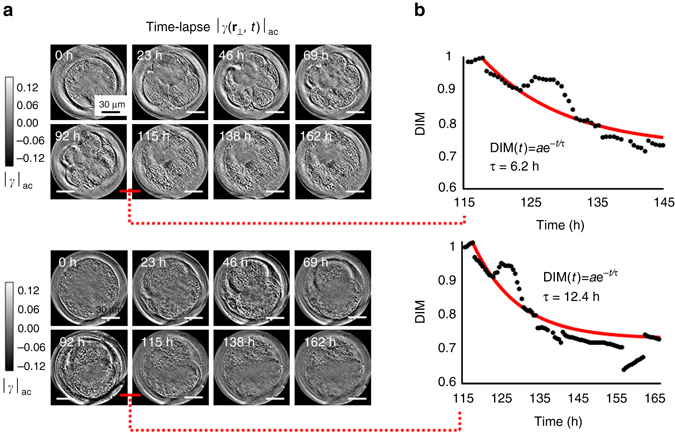
GLIM assay of embryo viability. **a** Bovine embryos were observed under regulated atmospheric conditions, with a 40×/0.75 NA objective and a fully open condenser. As outlined in “Methods”, |*γ*(**r**_⊥_,*t*)|_ac_ is a high-passed version of |*γ*|, such that |*γ*(**k**_⊥_,*t*)|_ac_ = |*γ*(**k**_⊥_,*t*)|*F*(**k**_⊥_) with *F*(**k**_⊥_) the filter function defined in Eq. (11). **b** The internal structure dynamics of embryos was quantified by using *DIM*, based on the time derivative of the stack. DIM shows a continuous decrease over several hours prior to the embryo’s exhaustion. The raw DIM profiles (*black dotted*) and their fitted profiles (*red*)

Intuitively, during periods of inactivity, the spatial distribution of ${\it{\gamma }}_i^\prime$ across the embryo is uniform, the histogram is narrow compared to periods of higher activity. We found that between the point of apparent normal, dynamic behavior and the one with a complete lack of dynamic behavior, there exists a continuous process that lasts many hours (Fig. 4a). This process is well captured by the intrinsic DIM quantity, as shown in Fig. 4b. Here, the *black dotted* profiles are the raw measurement of DIM as a function of time *t* for different embryos. We found that DIM decreases continuously over several hours until it reaches the point where the embryo motion is suppressed. Further, the DIM(*t*) profiles are well described by exponentially decay functions (*red profiles* in Fig. 4b) with time constants of 6.2 h and 12.4 h, respectively. Therefore, we anticipate that this intrinsic dynamic marker can potentially hold valuable viability prediction capability, beyond the current, morphology-based assays.

### Embryo tomography with GLIM

**Fig. 5:**
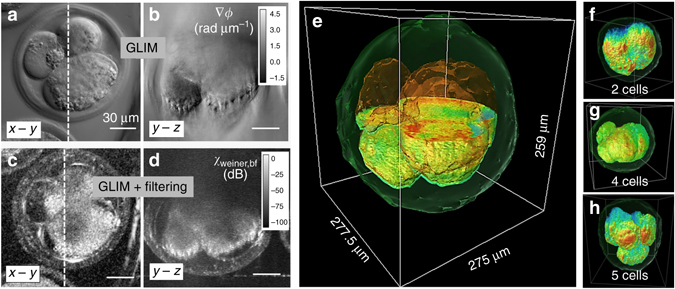
GLIM imaging for thick embryos. **a** *x*−*y* cross-section and **b** *y*−*z* cross-section of raw GLIM images in a bovine embryo. **c** *x*−*y* and **d** *y*−*z* cross-sections after spatial filtering. **e** A rendered embryo cut through the center to reveal internal structures. **f**–**h** Bovine embryos at different points in their development stages, as indicated

We obtained 3D GLIM stacks of bovine embryos at different development stages. We used a 63×/1.4 NA oil immersive objective at a transverse sampling rate of 10.8 pixel µm^−1^. The condenser aperture was fully opened to NA_con_ = 0.55 to maximize the depth sectioning and spatial resolution. The embryos were scanned in the axial dimension over an interval of (−120 µm, 120 µm) with a step of Δ*z* = 0.05 µm. Figure 5a and b shows the *x*−*y* and *x*−*z* cross-sections of the raw phase gradient, ∇*ϕ*. As a side note, in comparison with other QPI method, such as the Spatial Light Interference Microscopy (SLIM)^29^, GLIM is superior when imaging optically thick samples like embryos (Supplementary Note 9; Supplementary Fig. 8). The corresponding cross-sections of the GLIM tomogram after filtering are shown in Fig. 5c and d. More details on this filtering step can be found in the “Methods” section. The GLIM tomography, reveals various structures of the embryos, including their membranes, internal cells, gaps between the membrane of the cells, and their internal content, lipid droplets in each cell, as indicated in Fig. 5e. The *x*−*z* cross-sections further show the contact between the embryo and the underlying glass substrate (Fig. 5d), along with debris on the substrate.

Figure 5f–h shows the rendering results of three different embryos, consisting of two cells, four cells, and five cells, as indicated. One can see clearly how different cells of the embryo stack with respect to each other in 3D. The membranes of the embryos are manually segmented and displayed as transparent surfaces. Rendering videos of these embryos can be found in the Supplementary Movies 5–8.

## Discussion

In summary, we introduced GLIM, as a new QPI method, for 3D imaging of unlabeled specimens. GLIM has all the benefits of common-path white-light methods including nanometer path-length stability, speckle-free, and diffraction-limited resolution. At the smallest condenser aperture, GLIM gives exact values of the quantitative phase for thin samples. At the largest condenser aperture, GLIM can be used as a tomography method, allowing us to obtain time-lapse 3D information of thick samples. We demonstrated the success of GLIM on various samples, from beads, HeLa cells, to bovine embryos. We believe that this method will set an excellent foundation for other research projects and applications.

As a label-free method, GLIM can be applied to imaging live cells and thick samples nondestructively over broad temporal and spatial scales. This technique is not limited by photobleaching and phototoxicity commonly associated with fluorescence microscopy. Also, it provides excellent optical sectioning and obtains 3D information from unlabeled specimens. However, similar to other label-free images, GLIM lacks specificity. Therefore, we envision that GLIM and fluorescence techniques will co-exist and corroborate the advantages of specificity and noninvasiveness. This is completely feasible since GLIM operates on the same optical path as the fluorescence channels, allowing a seamless transition between the two modalities.

## Methods

### GLIM optical setup

The GLIM add-on module is mounted to the output camera port of a conventional DIC microscope. The measurements used in Fig. 1b were acquired with an Olympus IX70 microscope equipped with a 20×/0.65 NA objective. Subsequent measurements were conducted using an Axio Observer Z1 microscope with incubation system (Zeiss) under ×40/0.75 NA (420361-9910-000) and 63×/1.4 NA (420781-9910-0000) objectives. The GLIM module contains a polarizer before the camera and the SLM (Meadowlark) is positioned to match the DIC shear angle (45°). To ensure optimal modulation, the SLM was illuminated with filtered white light using a fluorescent emission filter with a central wavelength of *λ*_o_ = 624 nm and a bandwidth of Δ*λ* = 43 nm.

### Phase-gradient extraction from intensity images

The intensity image at modulation *ϕ*_*m*_ is given by

8 $${I_n}\left( {\bf{r}} \right) = I\left( {\bf{r}} \right) + I\left( {{\bf{r}} + \delta {\bf{r}}} \right) + 2\left| {\gamma \left( {{\bf{r}},\delta {\bf{r}}} \right)} \right|\cos \left[ {\Delta \phi \left( {\bf{r}} \right) + {\phi _n}} \right],$$

where Δ*ϕ* = *ϕ*(**r** + *δ* **r**)−*ϕ*(**r**) ≈ ∇(*ϕ*)*δ*r, the phase difference of interest, and ∇*ϕ* the gradient of the phase in the direction of the shift. The spatial shift *δ* **r** is the transverse displacement introduced by the DIC prism, estimated experimentally from measurements of the test samples. The quantity *γ*(**r**, *δ* **r**) is the mutual intensity, in other words, the temporal cross-correlation function between these two fields, evaluated at zero delay, *γ*(**r**, *δ* **r**) = 〈*U* ^*^(**r**)*U*(**r** + *δ* **r**)〉_*t*_. Combining the four intensity frames, we obtain the phase gradient as

9 $$\nabla \phi \left( {\bf{r}} \right) = \arg \left\{ {\left[ {{I_4}\left( {\bf{r}} \right) - {I_2}\left( {\bf{r}} \right)} \right],\left[ {{I_3}\left( {\bf{r}} \right) - {I_1}\left( {\bf{r}} \right)} \right]} \right\}/\delta r.$$

### DIC shear estimation

The lateral offset between the two DIC beams, *δ* *r*, relates the phase difference image, Δ*ϕ*, linearly to a quantitative phase gradient, ∇*ϕ*, via the following relation Δ*ϕ* = ∇*ϕδr*. Although this parameter is known to the microscope manufacturer (Zeiss, Olympus, etc.), to the best of our knowledge, it is not publicly listed. To estimate the spacing between the two beams, we matched the associated phase shift to a known calibration sample. In our procedure, we acquired a fine tomographic stack of a small object (300 nm bead), and performed line integration in the direction of the DIC gradient. As expected, the phase was always maximized at the plane of best focus. The peak of the integrated phase corresponds to the theoretical maximum phase shift due to the control structure, namely *φ* ^*^ = (2*πd* Δ*n*/*λ*_o_), where Δ*n* is the refractive index mismatch between the sample and the background, *λ*_o_ is the central wavelength, and *d* is the diameter of the bead. However, given a pixel dimension of *p* (µm), we also have another relationship ${\varphi ^*} = {\int}_{{x_{\rm{o}}}}^{{x^*}} {{\nabla _x}\varphi {\rm{d}}x} \approx {\int}_{{x_{\rm{o}}}}^{{x^*}} {\Delta \varphi {\rm{d}}x/\delta r} \approx p\mathop {\sum}\nolimits_{k = {k_{\rm{o}}}}^{{k^*}} {\Delta \varphi \left[ k \right]/\delta r} ,$ where *x*_o_, *x* ^*^ are locations of the background and somewhere inside the control structure. These locations correspond to pixel indices of *k*_o_ and *k* ^*^, respectively. Combining these two relations yields

10 $$\delta r = \frac{{p{\lambda _{\rm{o}}}\mathop {\sum}\limits_{k = {k_{\rm{o}}}}^{{k^*}} {\Delta \varphi \left[ k \right]} }}{{2\pi d\Delta n}}.$$

Using this formula, we estimated that the DIC prism shifts are 175 nm for 63×/1.4 NA and 345 nm for 40×/ 0.75 NA sliders. These values are in good agreement with reported literature^66^.

### 2D real-time interferometric reconstruction

The 2D image formation model in GLIM is shown in Supplementary Note 3, where we relate the measured phase difference Δ*ϕ* with the sample transmission, *T*. To fully automate the data acquisition for Δ*ϕ*, we developed a software platform capable of mechanical automation and real-time phase retrieval. Our image acquisition platform is designed to overlap the GLIM computation with the operation of the camera, SLM, and microscope. The software is developed in C++ using the Qt framework. The real-time reconstruction runs on three threads with the first thread responsible for triggering new camera frames and modulating the SLM. The second thread receives incoming images and transfers them to the graphics card. The third thread is used to display the GUI and render the resulting phase maps (Supplementary Fig. 2a). As we decouple the triggering thread from the data transfer and computation threads, variability in camera transfer rates does not slow down the acquisition.

To remove the phase slant found in commercial DIC microscopes (see ref. ^42^), we perform Fourier bandpass filtering. Here, we construct a filter function that does bandpass filtering as

11 $$F\left( {{{\bf{k}}_ \bot }} \right) = \left\{ {1 - \exp \left[ { - k_ \bot ^2/\left( {2\rho _{hi}^2} \right)} \right]} \right\}\exp \left[ { - k_ \bot ^2/\left( {2\rho _{lo}^2} \right)} \right],$$

where *ρ*_hi_ and *ρ*_hi_ define the bandwidth of the high-pass and low-pass filtering operations, respectively. Their values also depend on the system magnification. This operation eliminates the slow-varying oscillation in the GLIM images. The results of this operation are shown in the Supplementary Fig. 2b. To improve the SNR of the reconstruction, we match the mean intensities of the four frames by proportionally adjusting the exposure time. For example, the exposure time is eight times longer for the extinction frame (*π* modulation) compared to maximum brightness frame (0*π* modulation). The longer exposure is later compensated numerically in extracting the phase (Supplementary Fig. 2c). This operation results in an increased signal-to-noise ratio (SNR), thanks to a reduction in the phase noise deviation (Supplementary Fig. 2c).

Our GLIM system operates at 10 phase images per second with a rendering rate at 40 frames per second. As the computational portion is overlapped with acquisition, the rate-limiting factor in our system is the exposure time. Thus, longer exposure can be replaced by illumination with a brighter source. After acquiring individual images from different FOVs, we combine them together, forming a large mosaic to study large-scale dynamics. See Supplementary Note 4 and Supplementary Fig. 3 for implementation details on the image alignment and registration algorithm.

### Filtering method to improve depth sectioning of GLIM

To improve the optical sectioning, and push GLIM into a 3D imaging method, we removed the low-frequency (out-of-focus) components from our data using a high-pass filter. Steps of our methods are summarized in the Supplementary Fig. 9. First, we removed the DIC shading artifact using Wiener deconvolution^67^. The 3D point spread function of the system is given as $h\left( {\bf{r}} \right) = {{\rm{Im}}} \left[ {\left( {{\mu _i}{g^*}} \right)\left( {\bf{r}} \right) - \left( {{\mu _i}{g^*}} \right)\left( {{\bf{r}} - {{\hat {\bf x}}}\delta x} \right)} \right],$ (Supplementary Note 10; Supplementary Fig. 10), the transfer function is $\tilde h\left( {\bf{k}} \right) = 2i\sin \left( {{k_x}\delta x/2} \right)F\,\left\{ {{{\rm{Im}}} \left( {{\mu _i}{g^*}} \right)} \right\}\left( {\bf{k}} \right)$. As a side note, a measurement of the point spread function using a microbead can be found in the Supplementary Fig. 11. The Wiener deconvolution result of the susceptibility can be obtained in the frequency domain as

12 $${\tilde \chi _{{\rm{weiner}}}}\left( {\bf{k}} \right) = \frac{{ - 2i\sin \left( {{k_x}\delta x/2} \right)\delta x}}{{\beta _{\rm{o}}^2\left[ {4{{\sin }^2}\left( {{k_x}\delta x/2} \right) + \varepsilon } \right]}}{\rm F}\,\left[ {{\nabla _x}\phi } \right]\left( {\bf{k}} \right),$$

where *ε* is a small number, set to be 10^−4^ to avoid amplifying frequency components with small SNRs. To further improve the axial resolution, it is necessary to significantly suppress the low-frequency components in *χ*_weiner_(**r**). We achieve this by applying high-pass filtering in the *x*−*y* domain for each recorded *z*-image. In each dimension (*x* and *y*), a convolution with a finite-length impulse response (FIR), chosen as *h*_hp_ (*x*) = (0.25, −0.25, 0, −0.25, 0.25), is applied. The result of this high-pass filtering, *χ*_weiner, hp_(**r**), (Supplementary Fig. 9b) has most of the small transverse frequencies suppressed and, as a result, yields very good depth sectioning. Note that this high-pass filtering step can be combined with the Wiener deconvolution step since both are linear operators. Also, there is no need to perform any *z*-processing in our proposed method. This allows the processing to interlace with image acquisition. After filtering, we applied a log-compression transform to increase the contrast of the retained high-frequency components in the output image and normalized so that the largest signal is 0 dB. To reject the background noise, we keep signals with amplitude larger than −100 dB. Finally, to smooth the image and remove high-frequency oscillations in the image, we further apply bilateral filtering^68^ on the transformed results to obtain *χ*_weiner, bf_(**r**_⊥_,*z*) (Supplementary Fig. 9c). There are two passes of 2D bilateral filtering. In the first pass, bilateral filtering is applied to each 2D Wiener deconvolved image *χ*_weiner_ (**r**_⊥_,*z*) for each value of *z*. The second pass applies bilateral filtering on the stacked result of the first pass for each 2D image of the same lateral coordinate *x*. Owing to the similarity, we describe here the first one. For each value *z*, the bilateral results *χ*_weiner,bf_ (**r**_⊥_,*z*) is obtained from *χ*_*weiner*_ (**r**_⊥_,*z*) using

13 $${\chi _{{\rm{weiner}},{\rm{bf}}}}\left( {{{\bf{r}}_ \bot },z} \right) = \hskip17pc\\ \frac{{{\int\!\!\!\int} {{{\rm d}^2}{{{\bf{r}}'}_ \bot }{c_{\rm{r}}}\left( {{{\bf{r}}_ \bot },{{{\bf{r}}'}_ \bot }} \right){c_{\rm{s}}}\left[ {{\chi _{{\rm{weiner}},{\rm{bf}}}}\left( {{{\bf{r}}_ \bot },z} \right),{\chi _{{\rm{weiner}},{\rm{bf}}}}\left( {{{{\bf{r}}'}_ \bot },z} \right)} \right]{\chi _{{\rm{weiner}},{\rm{bf}}}}\left( {{{{\bf{r}}'}_ \bot },z} \right)} }}{{{\int\!\!\!\int} {{{\rm d}^2}{{{\bf{r}}''}_ \bot }{c_{\rm{r}}}\left( {{{\bf{r}}_ \bot },{{{\bf{r}}''}_ \bot }} \right){c_{\rm{s}}}\left[ {{\chi _{{\rm{weiner}},{\rm{bf}}}}\left( {{{\bf{r}}_ \bot },z} \right),{\chi _{{\rm{weiner}},{\rm{bf}}}}\left( {{{{\bf{r}}''}_ \bot },z} \right)} \right]} }},$$

where the radially symmetric Gaussian functions were used for the closeness function ${c_{\rm{r}}}\left( {{{\bf{r}}_ \bot },{{{\bf{r}}'}_ \bot }} \right) = \exp \left[ { - 0.5\left\| {{{\bf{r}}_ \bot } - {{{\bf{r}}'} \bot }} \right\|_2^2/\sigma _{\rm{r}}^2} \right]$ and ${c_{\rm{s}}}\left( {\chi ,\chi '} \right) = \exp \left[ { - 0.5\left\| {\chi - \chi '} \right\|_2^2/\sigma _{\rm{s}}^2} \right].$ Here, ${\left\| . \right\|_2}$ denotes the *l*_2_− norm. The coefficients, *σ*_r_, *σ*_s_ are chosen to determine the amount of filtering. In our case, they are set to *σ*_r_ = 2.2μm and *σ*_s_ = 3% of the maximum value of the input data *χ*_weiner,bf_ (**r**_⊥_,*z*), respectively. Clearly, the output of the post-processing has better depth sectioning compared to the input image. Different structures and materials, which are not visible in the raw input, appear nicely in the output.

### Data availability

The data that support the findings in this paper are available upon request.

## Electronic supplementary material

Supplementary Information

Supplemental video 1

Supplemental video 2

Supplemental video 3

Supplemental video 4

Supplemental video 5

Supplemental video 6

Supplemental video 7

Supplemental video 8

Supplemental video 9
