# Supplementary material for: Gradient light interference microscopy for 3D imaging of unlabeled specimens
Source: Nat Commun. 2017 Aug 8;8:210. doi: 10.1038/s41467-017-00190-7 (PMC5547102; doi:10.1038/s41467-017-00190-7)
Supplement: Supplementary file 1 — Supplementary Information [file 41467_2017_190_MOESM1_ESM.pdf]

File Name: Supplementary Information
Description: Supplementary Figures, Supplementary Notes, Supplementary Methods and Supplementary References.

File Name: Supplementary Movie 1
Description: GLIM imaging of a HeLa culture over 10 hours. The image sequence is composed of 16 x 20 mosaic tiles, each of 280 x 277 μm2 area with an acquisition performed every 16 minutes. The images were assembled with custom software, and encoded with FFmpeg.

File Name: Supplementary Movie 2
Description: Time-lapse rendering results of Hela cells from the filtered GLIM data. The cells were imaged using a 63x/1/4 NA objective over 7.7 hours. The whole volume of interest is imaged once every 22 minutes. In the axial dimension, it is scanned over a total depth of 28 μm with a step size of 0.07 μm. Also, to reject the noise and weak signals, only signals above -100 dB is used for rendering.

File Name: Supplementary Movie 3
Description: Time-lapse GLIM sequence of a developing embryo over a week period (one week, 40x/0.75 NA). To prevent focus drifts each embryo as places in a custom fabricated well, and the sequence was digitally cropped to remove the background (black area).

File Name: Supplementary Movie 4
Description: Time-lapse GLIM movie of a developing bovine embryo (9 hours, 63x/1.4 NA). Due to sample drift, the image sequence was digitally aligned. The sequence was then cropped to remove the background (black area).

File Name: Supplementary Movie 5
Description: A bovine embryo rendered using the filter GLIM data with upper half of each cell removed to see internal structures. The embryo was scanned using a 63x/1.4 NA objective. The condenser aperture was maximized to 0.55 NA. In the axial dimension, the embryo is scanned over a total distance of 240 μm with a step size of 0.05 μm. Normalized filtered GLIM data with amplitude larger than -100 dB was displayed. Surface rendering was used for membranes of individual cells and the whole embryo.

File Name: Supplementary Movie 6
Description: A bovine embryo with two cells rendered using the filter GLIM data. The embryo was scanned using a 63x/1.4 NA objective. The condenser aperture was maximized to 0.55 NA. In the axial dimension, the embryo is scanned over a total distance of 240 μm with a step size of 0.05 μm. Normalized filtered GLIM data with amplitude larger than -100 dB was displayed. Also, surface rendering was used for membranes of individual cells and the whole embryo.

File Name: Supplementary Movie 7
Description: A bovine embryo with four cells rendered using the filter GLIM data. The embryo was scanned using a 63x/1.4 NA objective. The condenser aperture was maximized to 0.55 NA. In the axial dimension, the embryo is scanned over a total distance of 240 μm with a step size of 0.05 μm. Normalized filtered GLIM data with amplitude larger than -100 dB was displayed. Also, surface rendering was used for membranes of individual cells and the whole embryo.

File Name: Supplementary Movie 8
Description: A bovine embryo with five cells rendered using the filter GLIM data. The embryo was scanned using a 63x/1.4 NA objective. The condenser aperture was maximized to 0.55 NA. In the axial dimension, the embryo is scanned over a total distance of 240 µm with a step size of 0.05 µm. Normalized filtered GLIM data with amplitude larger than -100 dB was displayed. Also, surface rendering was used for membranes of individual cells and the whole embryo.

File Name: Supplementary Movie 9
Description: Time-lapse imaging of embryos with SLIM (left) versus GLIM (right). The SLIM images suffer from poor contrast due to multiple scattering.

## Supplementary Figures

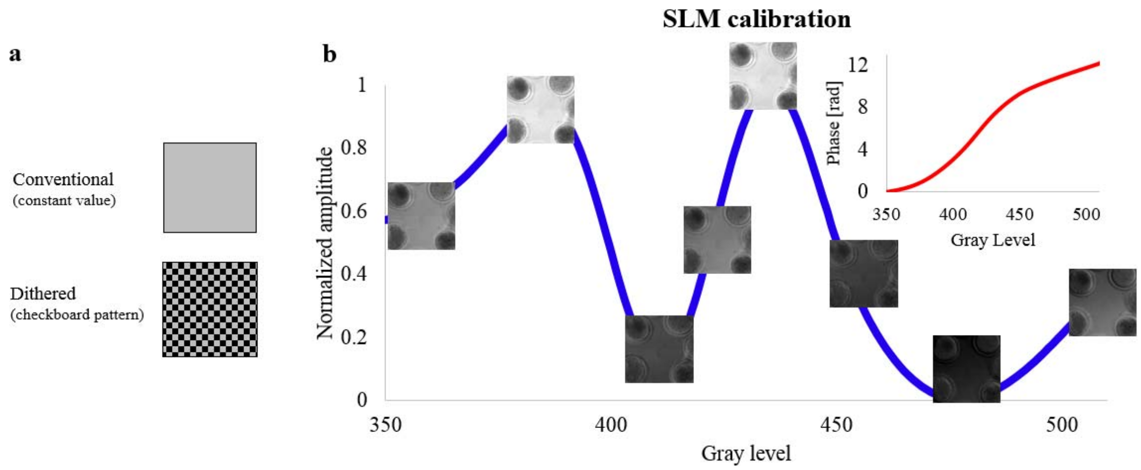


**Supplementary Figure 1 | SLM calibration**. (**a**) Conventional and checkerboard pattern (dithering) displayed on the SLM. We use the checker board pattern to obtain more than 256 gray levels. (**b**) Measured amplitude response (blue) and the reconstructed phase modulation curves (red) as function of the gray level. The insets along the blue curve show the contrast variation w.r.t different the gray level $g$.

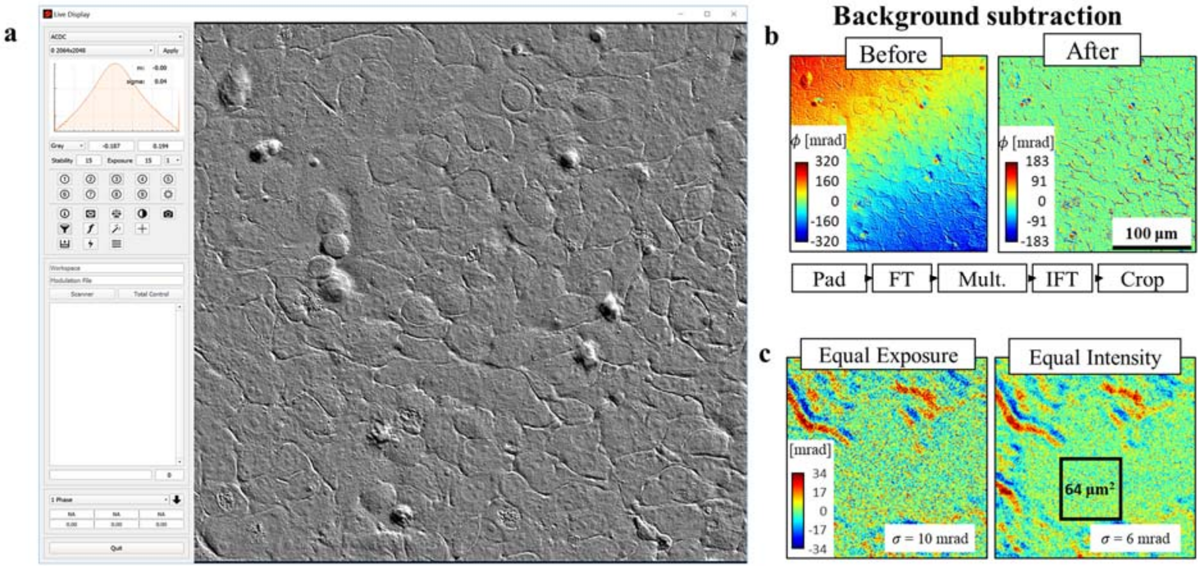


**Supplementary Figure 2 | Real-time interferometric reconstruction.** (**a**) Graphical user interface of our image acquisition software. (**b**) Image before and after background subtraction using Fourier bandpass filtering. (**c**) Image quality enhancement by adjusting the exposure time to match the intensity of four frames. The standard deviation of the noise decreased from 10 mrad to 6 mrad due to this step.

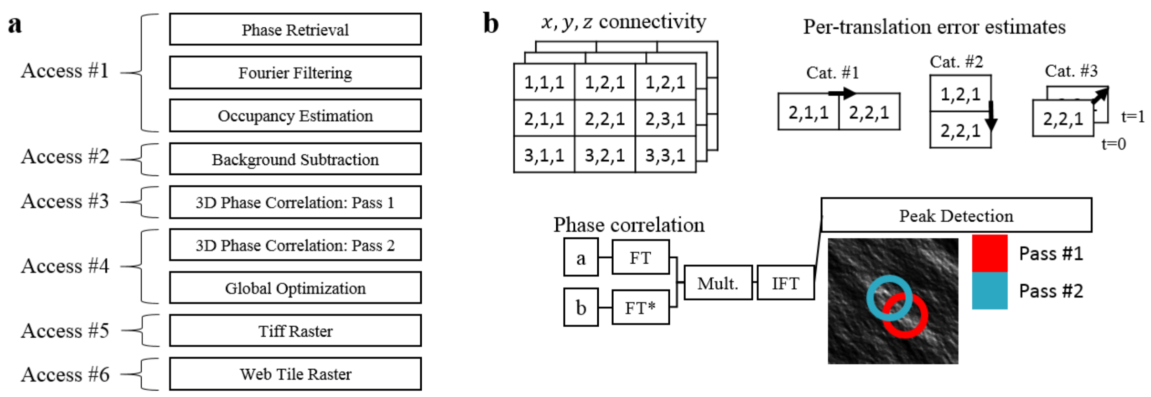


**Supplementary Figure 3 | Image assembly and registration.** (**a**) A summary of different steps to align and register small image tiles, and reconstruct a large FOV. Many data accesses are needed to produce the final mosaic. During the first two accesses, the same processing protocol is performed on each tile without taking into account there inter-relation. Access #3 & #4 calculate pair-wise relations between neighboring tiles. These relations are later used to solve an optimization problem to figure out exact locations of each tile in the final mosaic. Access #5 & #6 relates to data rendering and web archiving. (**b**) Detailed diagrams of the phase correlation step in Access #3 and the first part of Access #4. This phase correlation step extracts the pair-wise relation between neighboring tiles, which can be in space or time. To reduce the computational complexity, we deploy a GPU-based fast Fourier transform and its inverse. An extra peak detection step is also used to maximize the accuracy of the alignment.

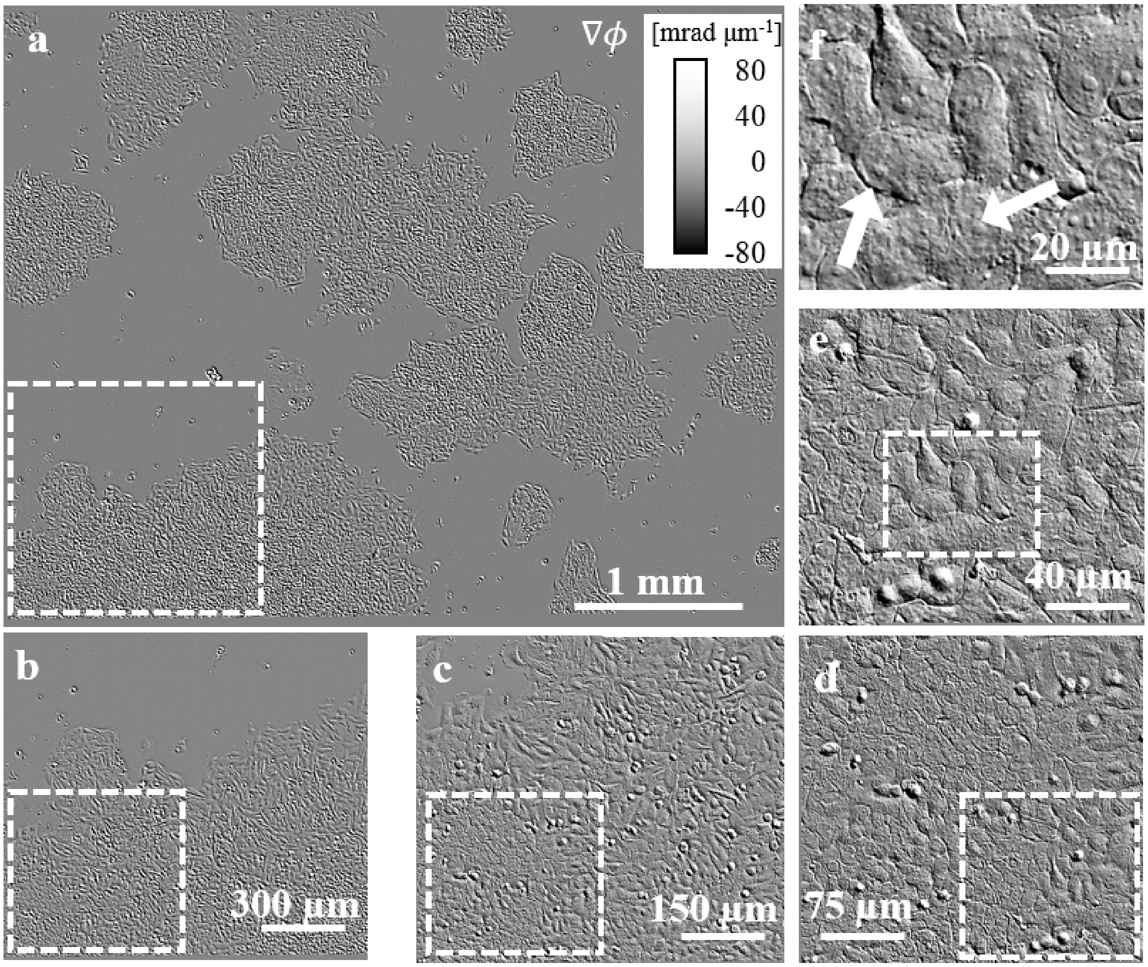


**Supplementary Figure 4 | GLIM images prepared for collaborative evaluation.** (**a**) A full FOV scan was performed over a period of 10 hours. It is composed of 16×20 mosaic tiles of size 280 ×277 μm$^2$ each. (**b**) – (**f**) Each image represents the selection show in the dashed box from the previous image. The scan was performed with submicron resolution to resolve cellular details for example, individual nucleoli, pointed by white arrows in (**f**).

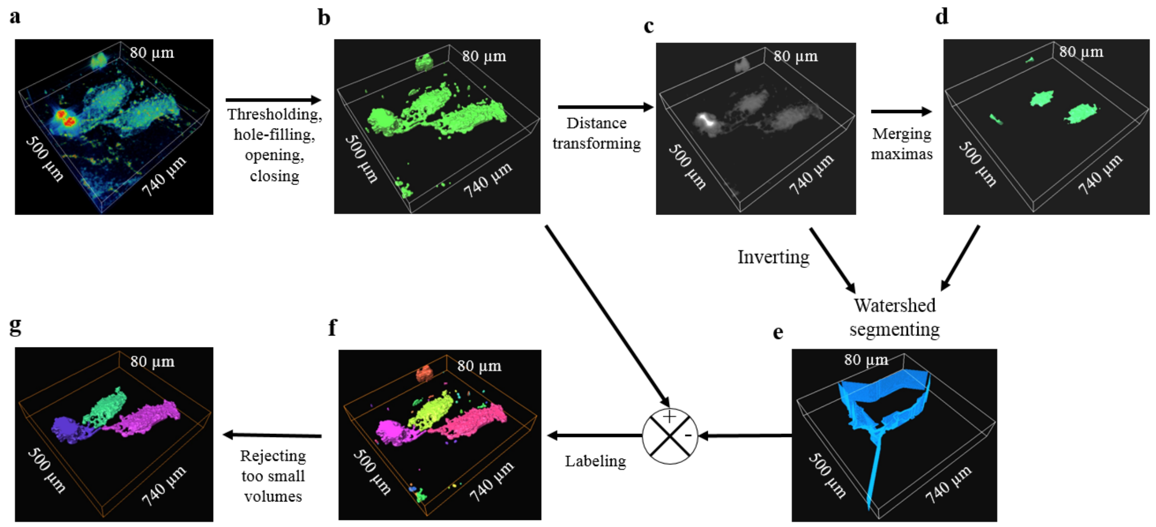


**Supplementary Figure 5 | Automatic segmentation scheme for HeLa cells.** (**a**) The GLIM filtered volumetric data is used as an input. (**b**) First, the data is thresholded and subsequently hole-filled. Small clumps of undesirable voxels due to thresholding are removed using the opening and closing operation. (**c**) **-** (**e**) The output binary data is segmented using the Watershed algorithm and distance transforming. (**f**) After segmenting, different cells are coded with different colors. (**g**) A final refinement step is applied to remove cells that is not fully contained in the imaging volume or too small voxel clumps.

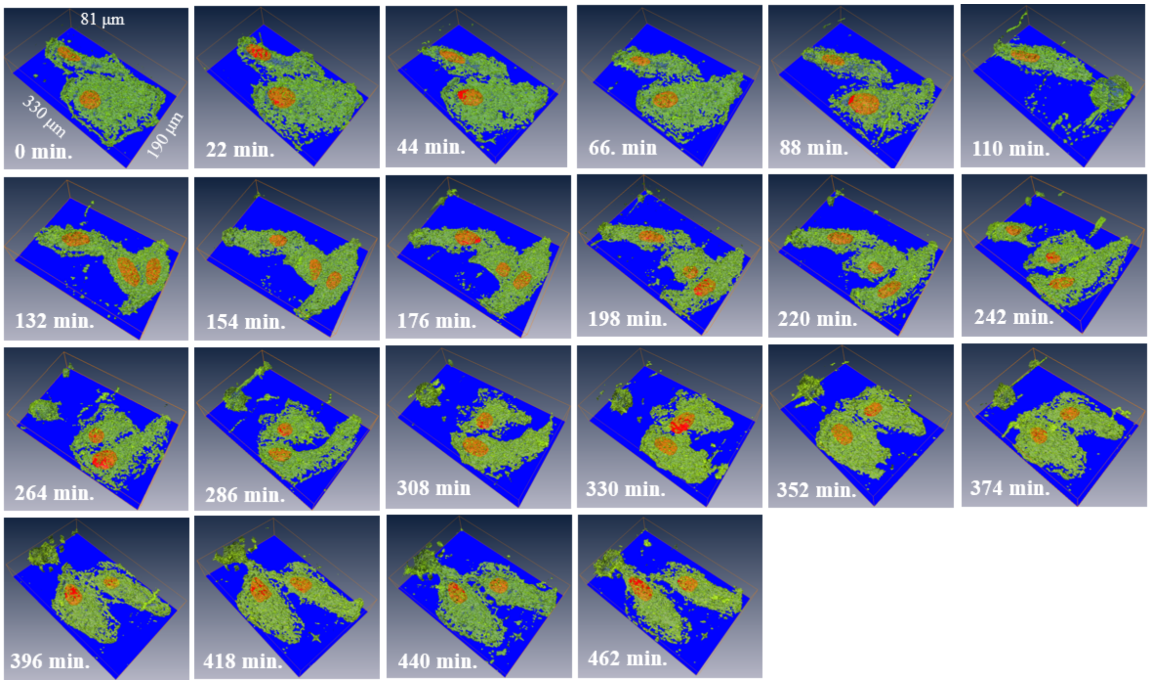


**Supplementary Figure 6 | Time-lapse GLIM imaging of small cells.** Rendering results of the HeLa cells over time, as indicated. In each panel, blue is for the substrate background. Orange covers segmented nuclei of the cells. Green is for their membranes.

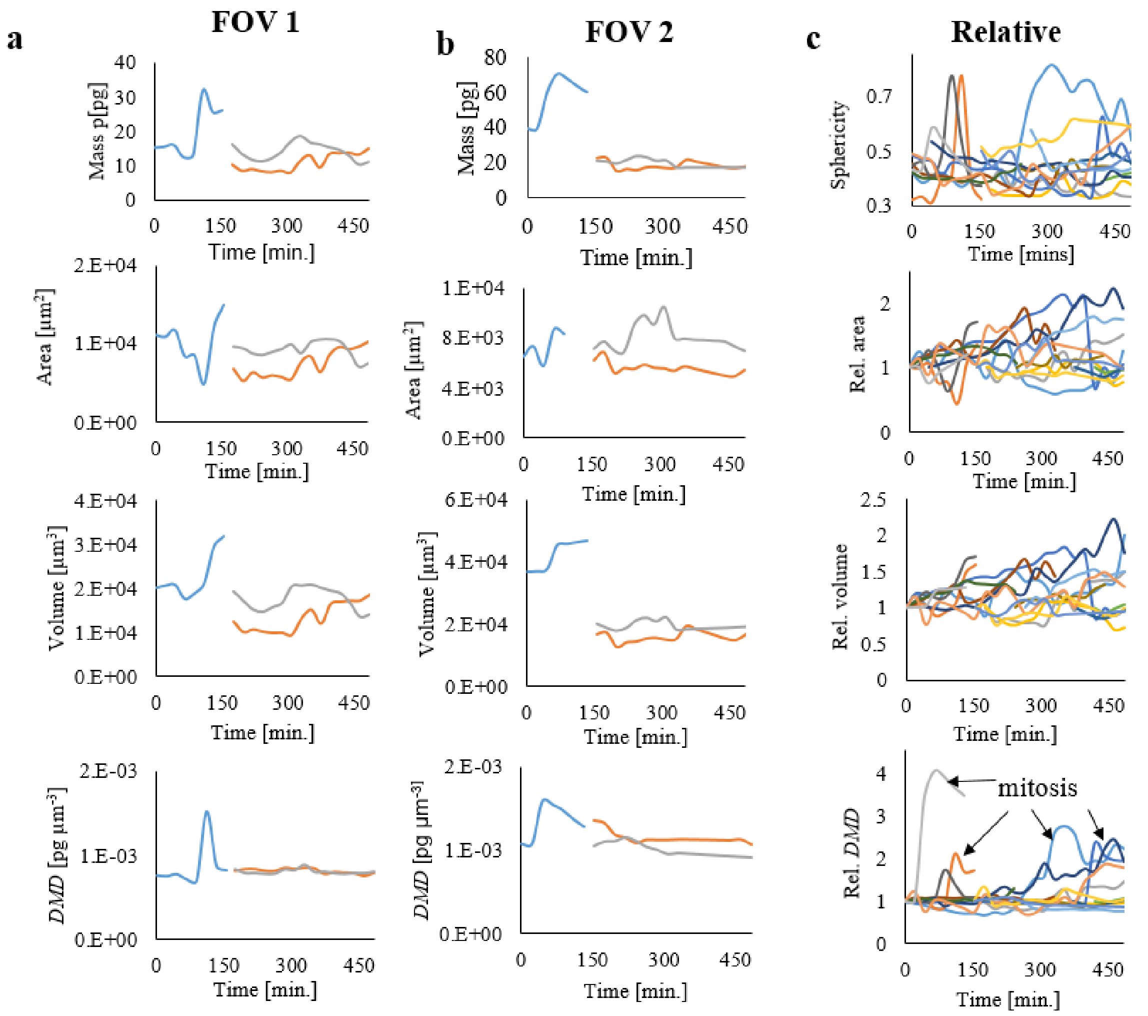


**Supplementary Figure 7 | Cell time-lapse measurement using GLIM.** (**a**) & (**b**) Measurements of the dry mass, surface area, volume and dry mass density of 17 HeLa cells from two different FOVs. (**c**) Measurements of the relative dry mass, relative surface area, relative volume, sphericity, and relative dry mass density of 17 HeLa cells. It can be seen that the dry mass density and sphericity increase significantly during mitosis. These time points are specified by the black arrows. In each panel, different colors code time profiles different cells. The same color is used across different panels if their coded profiles come from the same cell.

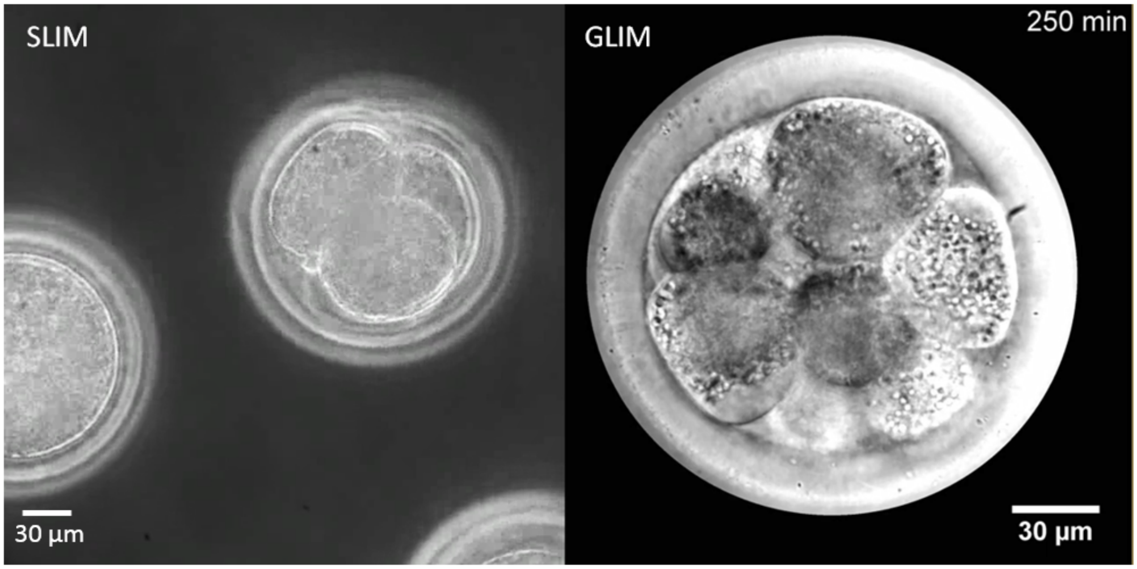

**Supplementary Figure 8 | Imaging embryos with SLIM (left) versus GLIM (right).** Here, bovine embryos are imaged with both techniques. While SLIM brings information at various depths into focus, GLIM is more depth selective. The middle (thickest) part of the embryo imaging using SLIM lost contrast due to multiple scattering. This is not the with the GLIM image. In summary, GLIM has better depth sectioning compared to SLIM.

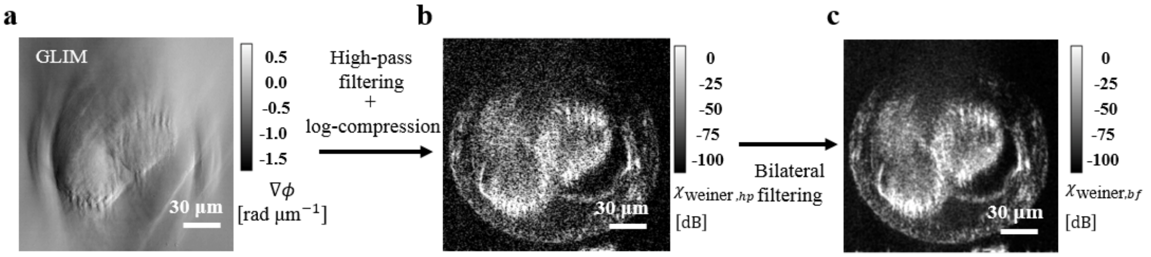


**Supplementary Figure 9 | Filtering scheme to improve depth sectioning.** The raw GLIM data (**a**) is filtered to suppress the low-frequency. The filter output is log-compressed, normalized, and thresholded to retain signals in the range of [-100, 0] dB (**b**). We further apply bilateral filtering to reduce the high-frequency oscillations due to the missing low-frequency and recover structures of the object (**c**).

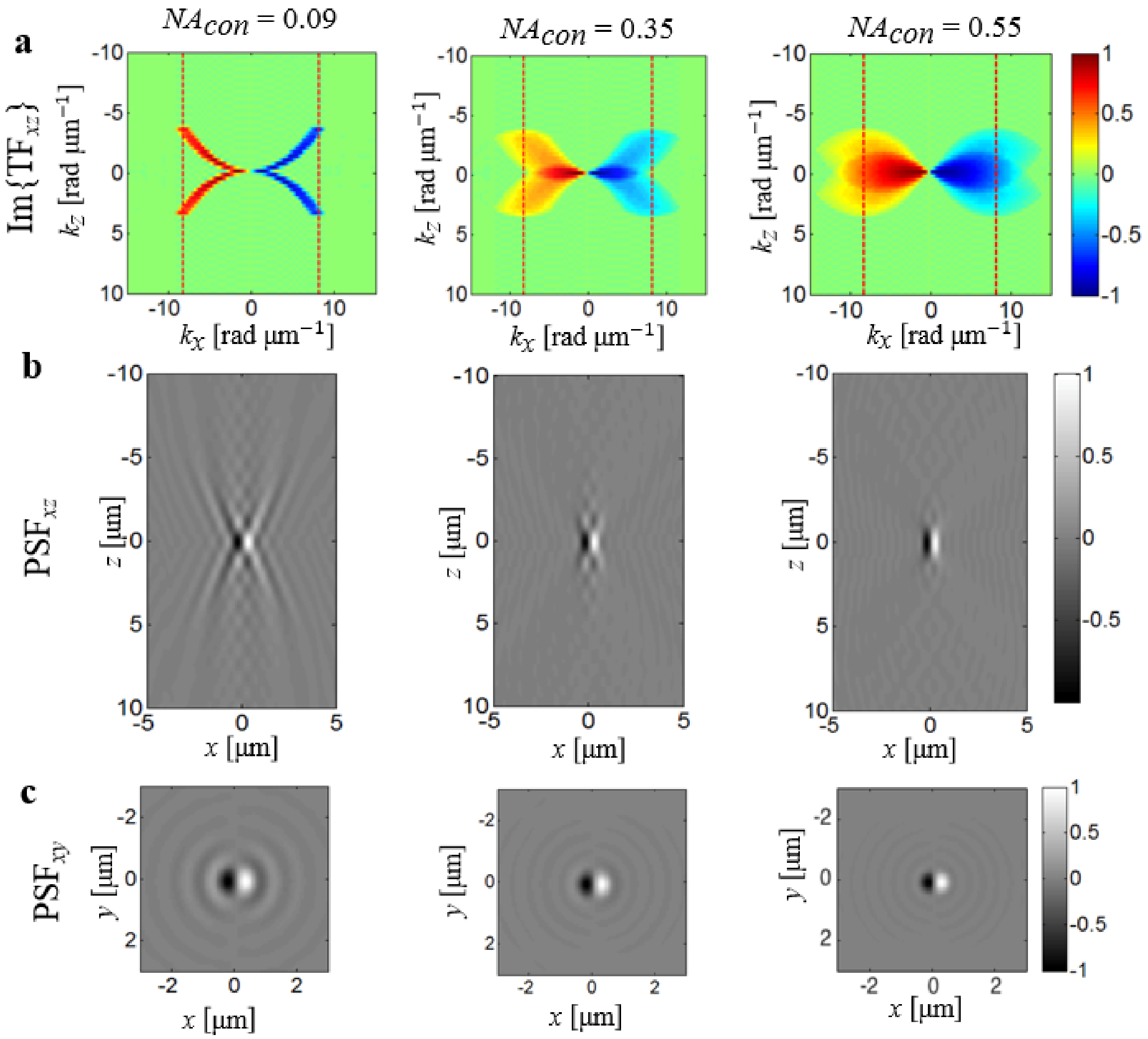


**Supplementary Figure 10 | Transfer function (TF) and Point Spread Function (PSF) of GLIM. (a)** The $k_x - k_z$ cross-sections of the TF. The dashed vertical lines denote the limiting numerical aperture of the microscope objective. (**b**) $x - z$ and ***(c)*** $x - y$ cross-sections of the PSF at different values of $NA_{con}$.

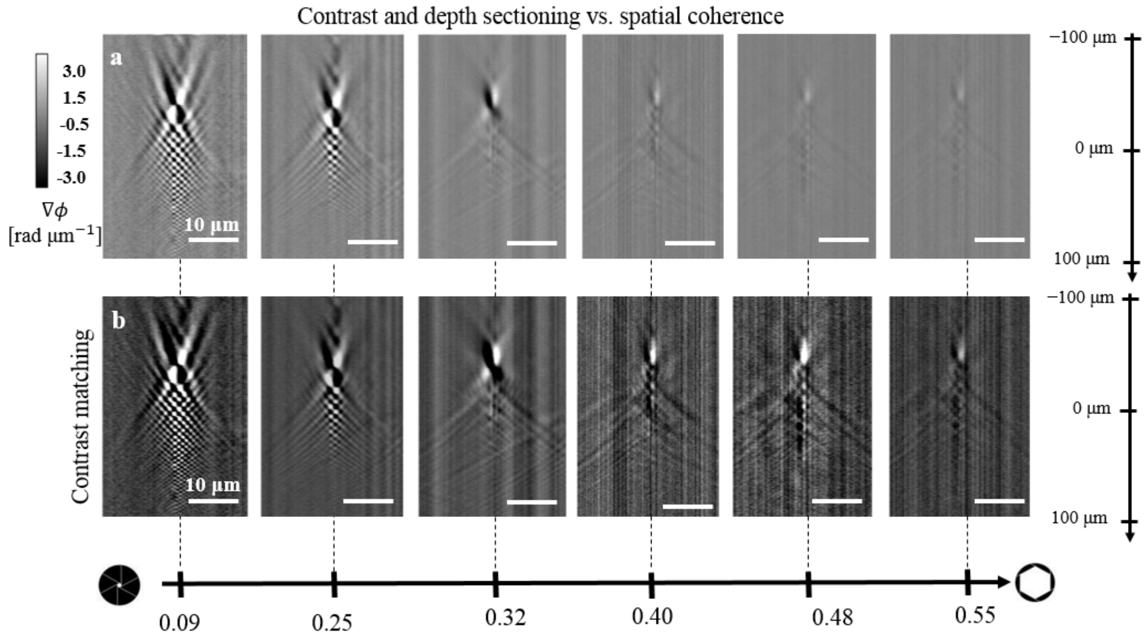


**Supplementary Figure 11 | Contrast and depth sectioning vs. spatial coherence.** (**a**) Raw $x-z$ cross-sections of the GLIM images of a 4.5 µm bead immersed in oil for different values of $NA_{con}$ with the same dynamic range applied. It can be seen that the contrast reduces in accordance with the spatial coherence of the illumination. (**b**) Contrast-enhance images of the images in (**a**). Clearly, the depth sectioning improves when the spatial coherence is smaller.

**SUPPLEMENTARY NOTE 1: Spatial light modulator (SLM) calibration**

We calibrate the SLM gray values vs. modulating phase by imaging the sample plane without the sample, i.e., $\Delta\phi(\mathbf{r})=0$. First, we acquire intensity images, $I_g$, with $g$ corresponds to the SLM grayscale values on an 8-bit dynamic range. Here, we further extend the resolution of the SLM modulation to one more bit by 1-bit dithering using a "checkerboard" pattern to the SLM (Supplementary Figure 1a). This means that neighboring pixels alternatively modulate the phase with gray levels of $g$ or $g+1$, depending on the parity of their indices. Mathematically, the modulation value set to the pixel $x$ is given as

$$S_{g+0.5}[x]=\begin{cases} g, & \text{if } x \text{ is even,} \\ g+1, & \text{if } x \text{ is odd.} \end{cases} \tag{1}$$

The intensity image $I_g$ (Supplementary Figure 1b) recorded by the camera for the grayscale value $g$ is

$$I_g = I_1 + I_2 + 2\sqrt{I_1 I_2}\cos\left[\phi(g)\right], \tag{2}$$

where $I_1$ is the intensity of the modulated field, $I_2$ is the intensity of the un-modulated field, and $\phi(g)$ is the phase modulation of interest. Using the experimentally measured $I(g)$ curve (Supplementary Figure 1b) for $g\in[0,255]$, we obtain $\cos[\phi(g)]=\left\{\left[I_g-\alpha\right]/\beta\right\}$, where $\alpha=\left\{\left[\max_g\left[I_g\right]+\min_g\left[I_g\right]\right]/2\right\}$, and $\beta=\left\{\left[\max_g\left[I_g\right]-\min_g\left[I_g\right]\right]/2\right\}$. Next, we use a Hilbert transform to obtain the complex analytic signal associated with the cosine signal, the imaginary part of which is

$\sin\left[\phi(g)\right] = H\left\{\left[I_g - \alpha\right]/\beta\right\}$. Here, $H(.)$ denotes the operator to compute the Hilbert transform from a real part of a complex analytic signal to get the imaginary part. Combining the sine and cosine signals, we obtain

$$\phi(g) = \arg\left\langle\left[I_g - \alpha\right]/\beta, H\left\{\left[I_g - \alpha\right]/\beta\right\}\right\rangle. \tag{3}$$

This $\phi(g)$ curve represents the desired SLM calibration. Note that there are many sets of four points that we can choose for the working phase shift. Experimentally, we choose the portion of the curve that is most linear (see Supplementary Figure 1b). From this curve, the $\pi/2$ sequence is chosen to meet two complementary criteria: maximum visibility and minimal phase error. To maximize the visibility, we evaluate several points around each amplitude maxima and choose the calibration where the steps are nearest to multiples of $\pi/2$. When multiple peaks are present, we prefer the peak most closely resembling a sinusoid (Supplementary Figure 1b), 2nd peak at $g$ =440), which corresponds to a linear $\phi_m(g)$ behavior. Our calibrated phase curve $\phi(g)$ is shown as the red profile in the inset of Supplementary Figure 1b.

**SUPPLEMENTARY NOTE 2: Pixel-to-pixel variation in GLIM modulation**

Our SLM is a XY model manufactured by Meadowlark Optics Inc. It has 512 × 512 pixels. Each pixel is controlled by an 8-bit grayscale value through the imaging computing. The reflected wavefront distortion is $\lambda/7$ at the central wavelength (RMS). However, thanks to the use of the common path geometry, both beams experience the same amount of distortion. Therefore, we do not expect this distortion has much detrimental effects on the image quality.

## SUPPLEMENTARY NOTE 3: Two-dimensional image formation in GLIM

Consider a thin sample with a transmission function $T(\mathbf{r}_\perp)$, the total incident field on it consists of two cross polarizations. They generate two sample fields on the camera plane of $U_1(\mathbf{r}_\perp) = \{[U_i(\mathbf{r}_\perp)T(\mathbf{r}_\perp)] \circledv_{\mathbf{r}_\perp} h_o\}(\mathbf{r}_\perp)$, and $U_2(\mathbf{r}_\perp) = \{[U_i(\mathbf{r}_\perp)T(\mathbf{r}_\perp - \delta\mathbf{r})] \circledv_{\mathbf{r}_\perp} h_o\}(\mathbf{r}_\perp)$, where $h_o$ is a point-spread function characterizing the limited-aperture diffraction of the objective. The temporal cross-correlation function at zero delays, $\tau = 0$, or the mutual intensity function of these two sample fields is

$$\begin{aligned}\gamma(\mathbf{r}_\perp, \delta\mathbf{r}) &= \langle U_1(\mathbf{r}_\perp) U_2^*(\mathbf{r}_\perp)\rangle_t \\ &= \int \Gamma_i(\mathbf{r}'_\perp - \mathbf{r}''_\perp) T(\mathbf{r}'_\perp) T^*(\mathbf{r}''_\perp - \delta\mathbf{r}) h_o(\mathbf{r}_\perp - \mathbf{r}'_\perp) h_o^*(\mathbf{r}_\perp - \mathbf{r}''_\perp) \mathrm{d}^2\mathbf{r}'_\perp\, \mathrm{d}^2\mathbf{r}''_\perp .\end{aligned}$$

(4)

Here, $\Gamma_i(.)$ is the mutual intensity function of the illumination at the sample plane. When the numerical aperture of the object, $\mathrm{NA_o}$, is large enough so that the spatial resolution is finer than any structure of interest in the sample, the PSF, $h_o$, can be approximated as $h_o(\mathbf{r}_\perp) \approx \delta^{(2)}(\mathbf{r}_\perp)$, simplifying Eq. (4) into

$$\gamma(\mathbf{r}_\perp, \delta\mathbf{r}) = \Gamma_i(\Delta\mathbf{r} = \mathbf{0}) T(\mathbf{r}_\perp) T^*(\mathbf{r}_\perp - \delta\mathbf{r}). \quad (5)$$

Consequently,

$$\Delta\phi(\mathbf{r}_\perp) = \arg[\gamma(\mathbf{r}_\perp, \delta\mathbf{r})] \approx \phi(\mathbf{r}_\perp) - \phi(\mathbf{r}_\perp - \hat{\mathbf{x}}\delta x), \quad (6)$$

with $\phi = \arg(T)$, which means that GLIM gives the correct phase difference of the sample irrespective of the coherence of the illumination. It is necessary to emphasize that the independence of the phase measurement to the spatial coherence for the system is obtainable for imaging system that has infinite spatial resolution. In practice, imaging systems are diffraction limited and, thus, there is a phase reduction on the measurement due to the spatial coherence. A special case happens when we close the condenser aperture to use the fully coherent illumination $\Gamma_i(\Delta\mathbf{r}) \to \text{const.}$ From Eq. (4), we have $\Delta\phi(\mathbf{r}_\perp) = \arg[\gamma(\mathbf{r}_\perp, \delta\mathbf{r})] \approx \arg[T \circledcirc_{\mathbf{r}_\perp} h_o](\mathbf{r}_\perp) - \arg[T \circledcirc_{\mathbf{r}_\perp} h_o](\mathbf{r}_\perp - \delta\mathbf{r})$. This result indicates that our measurement is the correct phase difference between diffracted versions of the diffracted wavefronts. Therefore, when the numerical aperture of the objective is finite, it is necessary to close the condenser aperture for a correct phase integration. When the condenser is not fully close, there is a reduction in the phase difference measurement. Our simulation and measurement shows that this reduction is smaller than that compared to other methods[1, 2] for the same numerical aperture of the objective.

## SUPPLEMENTARY NOTE 4: Assembling the two dimensional image mosaic

To assemble the time-lapse scans used in the Supplementary Fig. 2a, we developed an image alignment and registration algorithm, capable of efficiently handling very large FOV. Supplementary Fig. 3a summarizes different steps of this algorithm. We implemented it in PyCUDA to overlap computation with disk access, which is typically the rate-limiting factor. The algorithm proceeds by calculating the GLIM image (Access #1) and performing background correction. Additionally, understanding that empty regions have zero gradients, we estimate the occupancy of each tile by summing the magnitude of

the phase-difference images. The median phase value over the least occupied tiles is taken as a background measurement used to correct shared defects such as dust and scratches on the optics (Access #2).

The phase correlation algorithm (Access #3 & #4) generate a list of connections along the transverse dimensions and time (see Supplementary Figure 3b). This list is then sorted to improve performance by ensuring that mosaic tiles are read sequentially and relieved from computationally expensive steps such as disk access and the Fourier transform. For each pair of two neighboring tiles, $a(\mathbf{r}_\perp)$ and $b(\mathbf{r}_\perp)$, we find a displacement vector $\mathbf{r}_{\perp n} \in \Re^2$, such that $a(\mathbf{r}_\perp) = b(\mathbf{r}_\perp - \mathbf{r}_{\perp n})$. This vector is a maximizer of the correlation function between the two tiles, i.e., $\mathbf{r}_{\perp n(a,b)} = \arg\max_{\mathbf{r}_\perp}\left[(a \otimes b)(\mathbf{r}_\perp)\right]$ in our formulation. Here, the correlation function can be computed quickly using the Fast Fourier Transform (FFT) as

$$(a \otimes b)(\mathbf{r}_\perp) = \mathcal{F}^{-1}\left\{\mathcal{F}(a)\mathcal{F}(b)^*\right\}(\mathbf{r}_\perp). \tag{7}$$

We also masked the correlation function $(a \otimes b)(\mathbf{r}_\perp)$ with a searching window to refine dimensions of the searching area. To reduce the chance for secondary peaks, we performed a two-pass peak detection. In the first pass, a large search radius is used to gather the errors associated with each category of motion, see Supplementary Fig. 3b, red circle. In the second pass, the expected tile location is augmented with these per-category error estimates and the search radius is narrowed.

After estimating the optimal transverse displacement vector, $\mathbf{r}_{\perp n(a,b)}$, for each pair of neighboring tiles $(a,b)$ using the phase correlation algorithm, we do an additional global optimization step to find their best configuration of all tiles. Let $\mathbf{p}^\dagger[k] \in \Re^2$ be the optimal

location of $k^{th}$ tile and $\mathbf{p}^{\dagger} = \{\mathbf{p}^{\dagger}[1],...,\mathbf{p}^{\dagger}[N]\}$ be the best configuration of all the tiles to be optimized. We obtain $\mathbf{p}^{\dagger}$ as the solution of the following optimization problem

$$\mathbf{p}^{\dagger} = \arg\min_{\mathbf{p}} \sum_{k} \left\{ \sum_{j \in N(k)} \left\| \left( \mathbf{p}[k] - \mathbf{p}[j] \right) - \mathbf{r}_{\perp n(j,k)} \right\|^2 \right\}. \tag{8}$$

Here, $N(k)$ is the set of neighboring tiles of the tile $k^{th}$. The minimizer for the objective function in Eq. (8) is one that globally have location difference vector $\mathbf{p}[k]-\mathbf{p}[j]$ as close as possible to the estimated transverse displacement vector $\mathbf{r}_{\perp n(j,k)}$. We use an iterative approach to solve Eq.(8) using coordinates of tiles provided by the microscope, $\mathbf{p}_o[k]$, for the first iteration. After solving for $\mathbf{p}^{\dagger}$, we used it to generate floating point .tiff files for quantitative analysis (Access #5), and rendered them into the "mipmap" format used by Zoomify or TrakEM2 for easy access (Access #6).

Using this procedure, we showed zoomed-in images of the stitching results from all images in the whole FOV at different scales in Supplementary Fig. 4.

## SUPPLEMENTARY NOTE 5: Calculating $g(\mathbf{k}_{\perp}, \tau)$ from the phase gradient

The solution to the dispersion phase relation was computed directly from the phase gradient, $\nabla\phi$, which is possible because the it relates linearly to the dry mass density $\rho(\mathbf{r}_{\perp})$. Let us assume the shifting direction is in the $x-$direction, taking the two-dimensional Fourier transform of the phase gradient yields

$$\mathcal{F}_{\mathbf{k}_{\perp}}[\nabla\phi](\mathbf{k}_{\perp}) = k_x \tilde{\phi}(\mathbf{k}_{\perp}) = \xi k_x \tilde{\rho}(\mathbf{k}_{\perp}), \tag{9}$$

where $\xi$ is a constant, defined as $\xi = 2\pi\alpha/\lambda_o$ with $\lambda_o$ is central wavelength of the illumination and $\alpha$ 0.2 ml g$^{-1}$, the refractive index increment[3]. $\mathcal{F}_{\mathbf{k}_\perp}$ is the two-dimensional Fourier transform operator. Also, as an abuse of notation, we used $\tilde{\phi}(\mathbf{k}_\perp), \tilde{\rho}(\mathbf{k}_\perp)$ to denote the two-dimensional Fourier transform of the phase and the dry mass density evaluated at $\mathbf{k}_\perp$, respectively. Ignoring spatial frequencies along the $k_x = 0$ line, we can use the phase gradient $\nabla\phi$ to compute the autocorrelation function $g(\mathbf{k}_\perp, \tau)$ instead of the dry mass density $\rho$ using Eq. (9) as

$$g(\mathbf{k}_\perp,\tau) = \frac{\left\langle \tilde{\rho}^*(\mathbf{k}_\perp,t)\tilde{\rho}(\mathbf{k}_\perp,t+\tau)\right\rangle_t}{\left\langle \tilde{\rho}^*(\mathbf{k}_\perp,t)\tilde{\rho}(\mathbf{k}_\perp,t)\right\rangle_t} = \left.\frac{\left\langle \mathcal{F}^*_{\mathbf{k}_\perp}[\nabla\phi](\mathbf{k}_\perp,t)\mathcal{F}_{\mathbf{k}_\perp}[\nabla\phi](\mathbf{k}_\perp,t+\tau)\right\rangle_t}{\left\langle \mathcal{F}^*_{\mathbf{k}_\perp}[\nabla\phi](\mathbf{k}_\perp,t)\mathcal{F}_{\mathbf{k}_\perp}[\nabla\phi](\mathbf{k}_\perp,t)\right\rangle_t}\right|_{k_x\neq 0}. \quad (10)$$

## SUPPLEMENTARY NOTE 6: Automatic cell segmentation from GLIM filtered data

Automatic segmentation is crucial to obtain high-throughput, consistent, objective metrics on the cells during their development cycle. We describe our automatic segmentation procedure in Supplementary Fig. 5. An input stack of the raw GLIM data consists of three cells with one of them in mitosis is used for illustration (Supplementary Fig. 5a). First, the input is thresholded, hole-filtered, and morphologically transformed with the opening and closing with a $3\times3\times3$ structure element (Supplementary Fig. 5b). These steps eliminate spurious background noise, reduce the surface roughness, fill out gaps due to internal structures of the cells, and most importantly, generate a 3D binary map where a value of 1 are assigned to voxels inside the cells, and 0, otherwise. Then, the watershed algorithm[4] is used to produce separating barriers splitting the binary map into multiple regions corresponding to different cells. The watershed algorithm uses an inverted distance

map (Supplementary Fig. 5c) and some seeds generated as local maxima (Supplementary Fig. 5d) of the distance map. Here, the distance map is a scalar field where the value at each voxel is 0 if its value of the binary map is 0. On the other hand, when the binary is 1, the value at the voxel corresponds to the closest distance between it and the nearest boundary voxel. Next, the separating barriers (Supplementary Fig. 5e) are subtracted from the binary map and labels are given to connecting volumes in the resulting map (Supplementary Fig. 5f). Finally, we eliminate regions with volume measurement smaller than 900 $\mu m^3$ or 300,000 voxels, equivalently (Supplementary Fig. 5g).

**SUPPLEMENTARY NOTE 7: Dry mass estimation using GLIM**

Supplementary Fig. 6 shows time-lapse rendering results of the HeLa cells in the selected FOV mentioned in the main text. In this figure, orange is for the cell nuclei, green is for the membrane, and blue is for substrate background. Combining this FOV and others, we obtained several metrics over the cells during their development cycles.

R. Barer[5] showed that in two cases of serum albumin and serum globulin, the following relation holds with good accuracy $\alpha = \Delta n/C = \left[\left(n_p - n_s\right)/C\right]$. Here, $\alpha$ is the refractive index increment, $C$ is the number of grams of dry protein per 100 ml, and $n_p, n_s$ are the refractive indices of the protein and the solvent, respectively. Note that this relation holds in the 3D settings where these quantities are functions of the 3D coordinate $\mathbf{r}$. The quantity $C$ is the mass density of dry protein, calculated as $\left(\mathrm{d}m/\mathrm{d}V\right)(\mathbf{r})$, where $m$ is the total dry mass of the cell, and $V$ is its volume. Therefore,

$$m = \int_{\mathbf{r}\in V} \mathrm{d}m(\mathbf{r}) = \int_{\mathbf{r}\in V} \frac{\Delta n(\mathbf{r})\mathrm{d}V(\mathbf{r})}{\alpha(\mathbf{r})} = \int_{\mathbf{r}\in V} \frac{\Delta n(\mathbf{r})\mathrm{d}^3\mathbf{r}}{\alpha(\mathbf{r})}. \tag{11}$$

where the variation of $\alpha(\mathbf{r})$ is relatively small, $\alpha \approx 0.18 - 0.21$ ml g$^{-1}$, with a common value of $\bar{\alpha} = 0.20$ ml g$^{-1}$. However, the accurate determination of volume $V$ is very challenging. This obstacle hinders Eq. (11) from wide-spread use. A workaround was suggested by Barer by assuming that the refractive index is integrated along the axial dimension during imaging, i.e., $\Delta n(\mathbf{r}) = \Delta n(\mathbf{r}_\perp)$ to convert Eq. (11) into

$$m = \int_{\mathbf{r}_\perp \in S} \frac{\Delta n(\mathbf{r}_\perp) h(\mathbf{r}_\perp) \mathrm{d}^2\mathbf{r}_\perp}{\bar{\alpha}} = \int_{\mathbf{r}_\perp \in S} \frac{\varphi(\mathbf{r}_\perp) \mathrm{d}^2\mathbf{r}_\perp}{\bar{\alpha}\beta_o}, \tag{12}$$

where $\varphi(\mathbf{r}_\perp) = \beta_o \Delta n(\mathbf{r}_\perp) h(\mathbf{r}_\perp)$, and $S$ the effective projected area of the cell. Unfortunately, this assumption is not applicable to optically thick sample. Here, we show how to obtain the total dry mass directly from Eq. (12) given that $V$ is accurately provided by GLIM. First, we write this equation in terms of the susceptibility using the following approximation, $\Delta n(\mathbf{r}) \approx \left[\left(n_p^2(\mathbf{r}) - n_s^2\right)/(2n_s)\right] = \left[\chi(\mathbf{r})/(2n_s)\right]$. Eq. (12) yields

$$m = \frac{1}{2n_s\bar{\alpha}} \int_{\mathbf{r} \in V} \chi(\mathbf{r}) \mathrm{d}^3\mathbf{r} \approx \frac{\bar{\chi}V}{2n_s\bar{\alpha}}. \tag{13}$$

In Eq. (13), the refractive index of the surrounding media $n_s$ can be approximated to 1.33[6]. To obtain the average susceptibility $\bar{\chi} = \left(\int \chi(\mathbf{r}) \mathrm{d}^3\mathbf{r} / V\right) \approx \tilde{\chi}^{(3)}(\mathbf{k} = 0)$, we assume that the spatial spectrum of the object is preserved through time so that $\bar{\chi}$ can be estimated using the absolute value of the filtered data i.e. $\bar{\chi} = \gamma\left(\int \left|\chi_{hp}(\mathbf{r})\right| \mathrm{d}^3\mathbf{r} / V\right)$ up to a constant $\gamma$. The relative dry mass can be estimated without knowing this constant.

**SUPPLEMENTARY NOTE 8: Time-lapse measuring of mass, surface area, volume, and sphericity of HeLa cells**

Supplementary Figures 7a & b shows time-lapse measurements for absolute total dry mass ($m$), surface area $(A)$, volume $(V)$ and dry mass density ($DMD = m/V$) for two different FOVs. Each FOV has one parent cell and two daughter cells. The filtered data are used for automatic segmentation and dry mass extraction using procedures outlined above. These relative quantities of these metrics are computed by dividing their absolute values to those at the first time point, $t$=0 minute and shown in the Supplementary Fig. 7c. We further display the sphericity ($S$), defined as $S = \pi^{1/3}(6V)^{2/3}/A$, over time. Here, we show relative metrics obtained from 17 different cells randomly selected over all FOVs. Each curve corresponds to one individual cell. Different colors are used for different cells. The same color is used across different panels to code profiles come from the same cell. Our results show that, the total dry mass, volume, and surface area of all cells increase over the time during interphase. Meanwhile, the *DMD* is almost constant. However, when the cells enter mitosis, confirmed by maxima of the sphericity (Supplementary Fig. 7c), the surface area and volume reduce since the cells ball up forming spheroids. This fact results in an increase of the dry mass density during mitosis.

## SUPPLEMENTARY NOTE 9: GLIM vs. SLIM side-by-side comparison

The improvement that GLIM brings over other QPI methods, including SLIM, is most evident in the case of imaging, thick, multiple scattering specimens, such as bovine embryos[7]. The fundamental difference between SLIM and GLIM is in their interferometric geometry. In SLIM, the role of the reference field is played by the incident light. In multiple scattering specimens, this field is exponentially attenuated, resulting in a weak reference beam, thus, low-contrast interferogram. This explains the low-contrast image in the SLIM

channel. On the other hand, in GLIM, the two interfering beams are always equal in power because they are spatially shifted replicas of the same image field. Now, the two fields interfere with high contrast no matter how thick the specimen. Still, the multiple scattering background does tend to reduce the contrast. However, this background consists of incoherent superposition scattered waves, which does not significantly change during the phase shifting process. Consequently, the multiple scattering background is largely removed upon combing the four frames, which involves two by two frame subtraction. This mechanism is exemplified clearly in the GLIM images of embryos.

Because of the multiple cell structure, each containing a large number of lipid droplets, the bovine embryos generate strong multiple scattering. Supplementary Fig. 8 illustrates the significant improvement in contrast in GLIM vs. SLIM. This figure is a frame from Supplementary Movie 9.

**SUPPLEMENTARY NOTE 10: 3D image formation in GLIM**

As shown above, small values of $NA_{con}$ gives precise value of the phase gradient. In this section, we focus on the other regime of large $NA_{con}$, where the depth sectioning is best thanks to the maximum angular coverage. Under the 1st order Born approximation[8], two sample fields coming to the camera plane can be written as

$$\begin{aligned} U_1(\mathbf{r}) &= U_i(\mathbf{r}) - \beta_o^2 \left\{ \left[ U_i \chi \right] \circledcirc_{\mathbf{r}} g \right\}(\mathbf{r}), \\ U_2(\mathbf{r}) &= U_i(\mathbf{r}) - \beta_o^2 \left\{ \left[ U_i \chi(\mathbf{r} + \delta \mathbf{r}) \right] \circledcirc_{\mathbf{r}} g \right\}(\mathbf{r}). \end{aligned} \tag{14}$$

Here, $g(\mathbf{r})$ is the propagation kernel, defined by the microscope's objective given by[9] as $g(\mathbf{r}) \approx i\mathcal{F}_{\mathbf{k}_\perp}^{-1} \left\{ e^{iqz} circ\left[ k_\perp / (\beta_o NA_o) \right] \right\} / (2\bar{n}\beta_o)$. Here, $\beta_o$ is the wavenumber in free-space.

$\mathcal{F}_{\mathbf{k}_\perp}^{-1}(.)$ is the inverse two-dimensional Fourier transform operator, and $circ(x)$ is the circular (or "disk") function, defined as $circ(x)=1$ if $x \le 1$ and 0, otherwise. $(\mathbf{k}_\perp, q)$ is a 3D wavevector constrained by the dispersion relation as $q=\sqrt{\beta_o^2 - k_\perp^2}$. Finally, "$\circledcirc_{\mathbf{r}}$" is the three-dimensional convolution operator in the spatial domain and $\chi(\mathbf{r})$ is the susceptibility of the sample. Hence, the temporal cross-correlation function $\gamma(\mathbf{r},\delta\mathbf{r}) = \langle U_1(\mathbf{r}) U_2^*(\mathbf{r}) \rangle_t$ of the two fields, at zero temporal delay, $\tau = 0,$ becomes

$$\begin{aligned}\gamma(\mathbf{r},\delta\mathbf{r}) = \Gamma_i(\mathbf{r},\mathbf{r};0) - \beta_o^2 \int \Gamma_i(\mathbf{r},\mathbf{r}';0)\chi^*(\mathbf{r}')g^*(\mathbf{r}-\mathbf{r}')\mathrm{d}^3\mathbf{r}' \\ -\beta_o^2 \int \Gamma_i(\mathbf{r}'',\mathbf{r};0)\chi(\mathbf{r}''-\hat{\mathbf{x}}\delta x)g(\mathbf{r}-\mathbf{r}'')\mathrm{d}^3\mathbf{r}'',\end{aligned} \tag{15}$$

where $\Gamma_i$ is the mutual intensity function of the illumination, which only depends on the coordinate difference under the statistically homogeneous assumption[9, 10], namely, $\Gamma_i(\mathbf{r}_1,\mathbf{r}_2;0) = \Gamma_i(\mathbf{r}_1 - \mathbf{r}_2) = \int S_c(\mathbf{k}_\perp) e^{i[\mathbf{k}_\perp \cdot (\mathbf{r}_{1\perp} - \mathbf{r}_{2\perp}) + q(z_1 - z_2)]} \mathrm{d}^2\mathbf{k}_\perp$. Here, $S_c$ is the aperture intensity of at the condenser aperture plane. Using this property of $\Gamma_i$, Eq. (15) can be simplified to

$$\gamma(\mathbf{r},\delta\mathbf{r}) = \Gamma_i(\mathbf{0};0) - \beta_o^2 \left\{ \chi \circledcirc_{\mathbf{r}} \left[ (\Gamma_i g^*)(\mathbf{r}) + (\Gamma_i^* g)(\mathbf{r} - \hat{\mathbf{x}}\delta x) \right] \right\}. \tag{16}$$

Assuming a non-absorbing object with a real $\chi$, with a uniform unit-amplitude intensity distribution at the condenser aperture, with $g(\mathbf{r}) \approx i\mathcal{F}_{\mathbf{k}_\perp}^{-1}\left\{ e^{iqz} circ\left[ k_\perp / (\beta_o NA_o) \right] \right\} / (2\bar{n}\beta_o)$, and $\Gamma_i(\mathbf{r}) \approx \mathcal{F}_{\mathbf{k}_\perp}^{-1}\left\{ e^{iqz} circ\left[ k_\perp / (\beta_o NA_c) \right] \right\}$, it is clear that the function $(\Gamma_i g^*)$ are purely

imaginary. Therefore, $\left(\Gamma_i^* g\right) = -\left(\Gamma_i g^*\right) = \mathrm{Im}\left(\Gamma_i^* g\right)$. As a result,

$$\begin{aligned}\nabla_x \phi(\mathbf{r}) &= \arg\left[\gamma(\mathbf{r}, \delta\mathbf{r})\right] / \delta x = \beta_o^2 \arctan\left\{\chi \circledcirc_{\mathbf{r}} \mathrm{Im}\left[\left(\mu_i g^*\right)(\mathbf{r}) - \left(\mu_i g^*\right)(\mathbf{r} - \hat{\mathbf{x}}\delta x)\right]\right\} / \delta x \\ &\approx \beta_o^2 \left(\chi \circledcirc_{\mathbf{r}} \mathrm{Im}\left[\left(\mu_i g^*\right)(\mathbf{r}) - \left(\mu_i g^*\right)(\mathbf{r} - \hat{\mathbf{x}}\delta x)\right]\right) / \delta x,\end{aligned} \tag{17}$$

where $\mu_i(\mathbf{r}) = \Gamma_i(\mathbf{r};0) / \Gamma_i(\mathbf{0};0)$, the complex degree of spatial coherence of the incident field. Supplementary Figure 10a show a $k_x - k_z$ cross-section of the imaginary part of the computed transfer function (TF) of the GLIM system for different values of the $NA_{con}$. This TF is obtained by 3D Fourier transforming the Point Spread Function (PSF), $h(\mathbf{r}) = \mathrm{Im}\left[\left(\mu_i g^*\right)(\mathbf{r}) - \left(\mu_i g^*\right)(\mathbf{r} - \hat{\mathbf{x}}\delta x)\right]$, whose cross-sections are shown in Supplementary Figures 8b & c, respectively. Since the PSF is odd, the TF is purely imaginary. The absolute value of the TF vanishes at $k_x = 0$, since a constant signal in the x-direction is filtered out by the gradient operator. Therefore, there is a missing area around $k_x = 0$ not covered by the TF. This region of missing frequencies is similar to the "missing cone" problem known in diffraction tomography[11]. Resolving this area requires rotating the sample[12] or using additional priors, for example, smoothness constraint[13], [14]. Larger $NA_{con}$ reduces the size of the missing cone, allowing the system to record more frequency components. The dashed red lines in Supplementary Figure 10a show to the limiting numerical aperture of the objective. The larger the $NA_{con}$, the more transverse frequencies are captured by the TF. More importantly, around $NA_{con}/2$, more $k_z$ frequency bandwidth is captured with $NA_{con}$ = 0.55 compared to those with $NA_{con}$ = 0.09, which essentially means that depth sectioning improves when $NA_{con}$ increases. The Supplementary Figure

10b shows multiple $x-z$ cross-sections of the PSF for different values of $NA_{con}$. Clearly, the axial elongation of the PSF decreases for larger $NA_{con}$. The improvement in depth sectioning for increasing $NA_{con}$ also reduces the ringing effects since less non-specific information from one $z-$plane is propagated into neighboring planes, as shown by the $x-y$ cross-section in the Supplementary Figure 10c. Clearly, the diffraction ringing phenomenon is suppressed when $NA_{con}$ increases.

Supplementary Figure 11a shows $x-z$ cross-sections of GLIM measurements of 4.5 μm beads vs. $NA_{con}$. The microscope scanned the beads in the axial direction over an interval of [-100 μm, 100 μm] with a step size of $\Delta z = 0.05$ μm. Here, the same dynamic range is applied to all images to study the change in contrast versus $NA_{con}$. Supplementary Fig. 11b has all images contrast normalized to assess depth sectioning better. Clearly, reducing the coherence of the illumination by increasing $NA_{con}$ improves the depth sectioning of the GLIM measurement at the cost of contrast reduction. Such improvement in depth sectioning is crucial when measuring optically thick samples. As a side note, one may notice an inconsistent behavior in the axial dimension when comparing the simulated PSFs in Supplementary Figure 10b and the measured ones in Supplementary Figure 11. While those in the Supplementary Figure 10b are perfectly symmetric, those in Supplementary Figure 11 are not. This break of symmetry is due to the existence of a glass substrate that the bead is sitting on during our measurements. Therefore, we also do not expect the PSFs to match perfectly. While the ultimate use PSF in Supplementary Figure 10b is for reconstructing over the whole volumetric body of the sample, most of which is

not close to the glass substrate, that of Supplementary Figure 11 is for validating the effect of spatial coherence.

**Supplementary Methods**

1. HeLa cells culture

Freshly thawed HeLa cells were cultured in DMEM with 10% fetal bovine serum at 37 °C with 5% $CO_2$. Cells were seeded onto a 35mm glass bottom dish coated with Poly-D-Lysine, and were allowed approximately five hours to settle. Before imaging the cells, the media was replaced, as DIC lids were not readily available, the dish was filled to maximum volume with DMEM. The dish was imaged under full illumination with the plastic glass top removed.

2. Bovine embryo preparation

*Reagents and Media*

Unless otherwise stated, all reagents were purchased from Sigma-Aldrich (*USA*).
The IVF medium was Tyrode's modified medium[15] without glucose and bovine serum albumin (BSA), supplemented with 5.3 SI/mL heparin, 30 µM penicillamine, 15 µM hypotaurine, 1 µM epinephrine, and 1% bovine serum (BS)(B9433). The IVC medium consisted of Synthetic Oviduct Fluid (SOF) medium[16], with 30 µL $mL^{-1}$ essential amino acids, 10 µL $mL^{-1}$ non-essential amino acids, and 5 % bovine serum (BS).

3. *In vitro* embryo production

The matured oocytes were purchased from DeSoto Biosciences (Seymour, TN, USA). *In vitro* matured cumulus-oocyte-complexes (COCs) were washed and transferred, 20-30 per

well, into 300 µL of IVF medium covered with mineral oil. Frozen sperms from a bull previously tested for IVF were thawed at 37 °C for 40 sec and selected by centrifugation (25 min at 300 X g) on a Percoll discontinuous gradient (45–80%). The pellet was reconstituted into 2 mL of IVF medium and centrifuged twice, at 160 and 108 X g for 15 min. The pellet was diluted with IVF medium and added to the fertilization wells at the concentration of 1 x $10^6$ sperm $mL^{-1}$. Gametes were co-incubated for 20 hours at 39 °C, in 5% $CO_2$ in the air, after which presumptive zygotes were vortexed for 2 minutes to remove cumulus cells in HEPES-TCM with 5% BS, washed twice in the same medium, and transferred into micro-wells of 220 µm[17]. The volume of the drop that covered the microwells was 50 µL of SOF. Zygotes were incubated in a humidified mixture of 5 % $CO_2$, 6 % $O_2$ and 88 % $N_2$ in air at the temperature of 39 °C.

### *4.* Microwell preparation for embryo imaging

The microwells are composed of PDMS and are made using soft lithography techniques. The polymer was mixed with its corresponding hardener, in a 10:1 ratio by weight, to create the PDMS mixture. Air bubbles were induced in the PDMS during mixing. The air was removed (degased) by placing the PMDS in an evacuated environment (desiccation chamber) for 45 minutes. After degassing, PDMS was placed on a hot plate at 60 °C for 4 hours.

**Supplementary References:**


1. Edwards C*, et al.* Effects of spatial coherence in diffraction phase microscopy. *Opt Express* **22**, 5133-5146 (2014).

2. Nguyen TH, Edwards C, Goddard LL, Popescu G. Quantitative phase imaging with partially coherent illumination. *Opt Lett* **39**, 5511-5514 (2014).

3. Barer R, Cole AR, Thompson HW. Infra-red spectroscopy with the reflecting microscope in physics, chemistry and biology. *Nature* **163**, 198-201 (1949).

4. Vincent L, Soille P. Watersheds in Digital Spaces - an Efficient Algorithm Based on Immersion Simulations. *Ieee T Pattern Anal* **13**, 583-598 (1991).

5. Barer R. Interference microscopy and mass determination. *Nature* **169**, 366-367 (1952).

6. Popescu G. *Quantitative phase imaging of cells and tissues*. McGraw-Hill (2011).

7. Wang Z*, et al.* Spatial light interference microscopy (SLIM). *Opt Express* **19**, 1016-1026 (2011).

8. Born M, Wolf E. *Principles of optics: electromagnetic theory of propagation, interference and diffraction of light*. CUP Archive (1999).

9. Nguyen TH, Edwards C, Goddard LL, Popescu G. Quantitative phase imaging of weakly scattering objects using partially coherent illumination. *Opt Express* **24**, 11683-11693 (2016).

10. Streibl N. Three-dimensional imaging by a microscope. *JOSA A* **2**, 121-127 (1985).

11. Sung Y, Choi W, Fang-Yen C, Badizadegan K, Dasari RR, Feld MS. Optical diffraction tomography for high resolution live cell imaging. *Opt Express* **17**, 266-277 (2009).

12. Choi W*, et al.* Tomographic phase microscopy. *Nat Methods* **4**, 717-719 (2007).

13. Su JW, Hsu WC, Chou CY, Chang CH, Sung KB. Digital holographic microtomography for high-resolution refractive index mapping of live cells. *J Biophotonics* **6**, 416-424 (2013).

14. Sung Y, Dasari RR. Deterministic regularization of three-dimensional optical diffraction tomography. *J Opt Soc Am A Opt Image Sci Vis* **28**, 1554-1561 (2011).

15. Parrish JJ, Susko-Parrish JL, Leibfried-Rutledge ML, Critser ES, Eyestone WH, First NL. Bovine in vitro fertilization with frozen-thawed semen. *Theriogenology* **25**, 591-600 (1986).

16. Tervit HR, Whittingham DG, Rowson LE. Successful culture in vitro of sheep and cattle ova. *J Reprod Fertil* **30**, 493-497 (1972).

17. Krisher RL, Wheeler MB. Towards the use of microfluidics for individual embryo culture. *Reprod Fertil Dev* **22**, 32-39 (2010).
